# Current Insights of the Potential Plant Bioactive Compounds on Modulating the mTOR Signaling Pathway in Regulating Oncological Disorders

**DOI:** 10.1002/ptr.70051

**Published:** 2025-07-27

**Authors:** Sonali Khanal, Manjusha Pillai, Md. Shimul Bhuia, Muhammad Torequl Islam, Onkar Sharma, Rachna Verma, Eugenie Nepovimova, Kamil Kuca, Dinesh Kumar, Raihan Chowdhury

**Affiliations:** ^1^ School of Bioengineering and Food Technology Shoolini University of Biotechnology and Management Sciences Solan India; ^2^ Department of Pharmacy Bangabandhu Sheikh Mujibur Rahman Science and Technology University Gopalganj Bangladesh; ^3^ Pharmacy Discipline Khulna University Khulna Bangladesh; ^4^ School of Biological and Environmental Science Shoolini University of Biotechnology and Management Sciences Solan India; ^5^ Centre of Advanced Innovation Technologies VSB – Technical University of Ostrava Ostrava‐Poruba Czech Republic; ^6^ Faculty of Science University of Hradec Kralove Hradec Kralove Czech Republic; ^7^ Biomedical Research Center University Hospital Hradec Kralove Hradec Kralove Czech Republic; ^8^ Center for Basic and Applied Science, Faculty of Informatics and Management University of Hradec Kralove Hradec Kralove Czech Republic

**Keywords:** apoptosis, autophagy, mTOR signaling pathway, oncology, pharmacology, plant bioactive compounds

## Abstract

Deregulation of the mechanistic target of the rapamycin (mTOR) pathway plays an important role in cancer genesis and progression, making it an attractive target for cancer treatment. Disrupting the mTOR pathway contributes to uncontrolled cell growth, increased proliferation, and enhanced cell survival, all of which are hallmarks of cancer. The findings imply that the deregulation of the mTOR pathway in cancer provides a compelling basis for therapeutic treatments. Phytochemicals such as flavonoids and polyphenols have been shown to play a key role in suppressing different kinases implicated in PI3K/AKT/mTOR signaling cascades. A thorough study of the molecular connections between bioactive substances, mTOR signaling, and autophagy activation can lead to the creation of personalized treatments that work better and have fewer side effects. Finding out how important it is to target the mTOR pathway and how to use bioactive chemicals from plants to treat cancer are both important discoveries that could lead to more precise medicine and the development of effective cancer drugs. Finally, targeting mTOR pathways using plant‐derived chemicals may be a promising therapy strategy for cancer. This review tries to summarize putative plant bioactive chemicals that act on malignancies via mTOR signaling pathways. Furthermore, the paper discusses the role of the mTOR pathway in cancer, specifically its influence on cell growth, proliferation, and survival.

## 1 Introduction

A crucial regulator of cell growth and metabolism, the mammalian target of rapamycin (mTOR) is a highly conserved protein kinase (Boutouja et al. 2019). It is an extensively conserved enzyme that governs crucial cellular processes like growth regulation, autophagy, and metabolism. This protein kinase is a common kind of enzyme that specifically targets the amino acids serine and threonine. It belongs to a group of kinases called phosphoinositide 3‐kinase‐related kinases (PI3K), which are found in all eukaryotic organisms. This kinase is located on a chromosome (Girodengo et al. 2022; Jhanwar‐Uniyal et al. 2024). Since it was discovered in the early 1970s, rapamycin, also produced by *Streptomyces hygroscopicus* , fundamentally targets mTOR (Brown et al. 1994). T regulatory cells, tumor‐associated macrophages (TAMs), CD4, CD8, myeloid‐derived suppressor cells (MDSCs), endothelial cells, and cancer‐associated fibroblasts (CAF) have all been shown to be modulated by mTORC1/2 in various studies (Thomson et al. 2009; Weichhart et al. 2015). Additionally, through TAMs, T cells, activation, and the development of antigen‐presenting cells, mTOR is connected to the control of the immune system (Mafi et al. 2022). Numerous physiological processes, including metabolism, motility, transcription, apoptosis, autophagy, cell cycle progression, and differentiation, are regulated by the PI3K/AKT/mTOR signaling pathway (Saxton and Sabatini 2017). It has been found that several pharmaceuticals, both natural and artificial, are effective in inhibiting mTOR and regulating the growth of cancer. Apigetron, dihydromyricetin, piperlongumine, thymoquinone, glycyrrhizic acid, cryptotanshinone, cannabisin B, licochalcone A, and curcumin are a few of the compounds that have demonstrated promise as anticancer medications (Kim et al. 2020). Herbal medications are one of the most trustworthy sources of phytonutrients, and they have the potential to be a natural therapy and a green alternative to the widely used radioactive treatment for numerous cancers. Worldwide recognition has been accorded to these conventional herbal remedies for their anti‐proliferative, anti‐angiogenic, anti‐inflammatory, anti‐oxidant, anti‐mutagenic, and anti‐invasive characteristics (Yu et al. 2013). Around 35,000 bioactive chemicals derived from plants, seaweed, and other sources have been identified to inhibit pathogenic signaling linked with cancer start, with no detrimental or adverse effects (Tao et al. 2018).

Alkaloids, terpenoids, quinones, saponins, flavonoids, volatile oils, and phenolics, for example, have a possible cytotoxic effect against colorectal cancer cells while posing fewer dangers and side effects (Gupta et al. 2022). Phytochemicals such as flavonoids and polyphenols have been shown to inhibit different kinases implicated in the PI3K/AKT/mTOR signaling cascades (George et al. 2017). Traditional herbal remedies, on the other hand, are not widely used due to a number of important issues, including the poor solubility and bioavailability of these substances. Other reasons for plant‐based medications include drug synergism, intellectual property difficulties, variety in extraction processes and sources, and lack of drug‐likeness (Esmeeta et al. 2022). The dysregulation of the PI3K/AKT/mTOR pathway is recognized as a key driver of cancer growth and progression. PI3K/AKT/mTOR pathway abnormalities are present in about 50% of malignancies and are the most frequently activated pathways in human cancer. Subsequent investigation has shown that mTOR is often active in neoplasms and governs cancer cell metabolism by regulating crucial metabolic enzymes. As a result, it has been focused on as a potential therapy for cancer. Furthermore, rapamycin hinders the growth of endothelial cells and safeguards against the worsening of the condition by the use of drug‐eluting stents administered into the artery (Glaviano et al. 2023; Zhang et al. 2023). Furthermore, monitoring mTOR activity is critical in cancer care. The status of mTOR signaling gives important information about disease progression and therapy response. Several methods are used to monitor mTOR in cancer, including molecular profiling, biomarker analysis, imaging techniques, and so on. Techniques like liquid biopsy and biosensing have recently emerged, providing a better assessment of mTOR monitoring. These techniques aid in identifying abnormal mTOR activity, predicting therapy effects, and guiding therapeutic decisions (Meric‐Bernstam and Gonzalez‐Angulo 2009; Bouquier et al. 2020; Moldogazieva et al. 2021; Marafie et al. 2024).

This review study discusses the effects of plant bioactive compounds on altering mTOR signaling and their possible use in cancer treatment. It also outlines the methods that are currently available for detecting mTOR activity. It investigates the role of mTOR in various oncological illnesses as well as how plant bioactive substances can control its action. It also examines the potential for combining natural therapies with a nanotechnology‐mediated inflammatory cascade to treat a variety of chronic and acute disorders. The study highlights the importance of knowing and monitoring mTOR in cancer treatment throughout the discussion and investigates various methodologies and procedures used for this aim.

### 1.1 mTOR Signaling Pathway in Cancer

**FIGURE 1:**
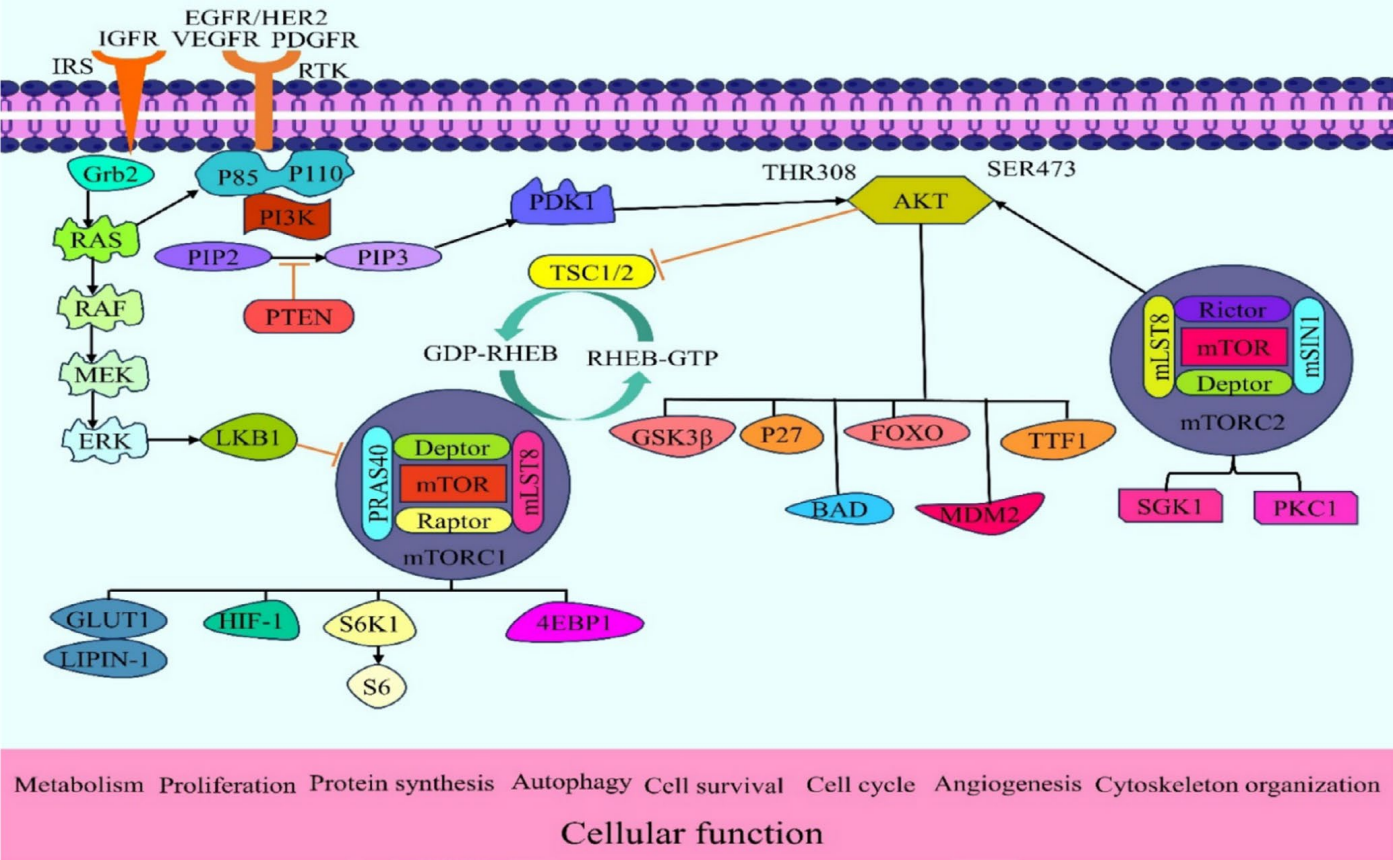
mTOR signaling pathway in cellular process and regulation of various cellular functions.

Protein kinase mTOR is divided into two signaling complexes, mTOR complex 1 (mTORC1) and mTOR complex 2 (mTORC2), and is a member of the phosphoinositide‐3‐kinase (PI3K) family (Laplante and Sabatini 2012). Though they phosphorylate distinct downstream substrates and exhibit varying biological functions, these two protein kinases share the same catalytic‐TOR component. In addition, there are three equivalents of mTORC1: mTOR, raptor (mTOR‐associated regulatory protein), and mLST8 (mammalian lethal protein 8 with Sec13, also referred to as GL). It also contains two inhibitory subunits, PRAS40 (proline‐rich AKT substrate of 40 kDa) and DEPTOR (mTOR‐interacting protein including DEP domain). Proton‐1/2 (found with rictor protein composition‐1/2), mSIN1 (mammalian stress‐activated protein kinase interaction protein 1), mTOR, mLST8, rictor (an mTOR‐independent raptor partner), and DEPTOR comprise mTORC2 (Battaglioni et al. 2022). While mTORC2 phosphorylates AKT on the primary phosphorylation site at Ser473, mTORC1 activity can be determined by examining the phosphorylation of ribosomal subunit S6 or the direct downstream target ribosomal subunit S6K1 on Thr389. AKT is activated by PI3K, and this leads to the phosphorylation of mTOR, which enhances cell survival and proliferation. VEGF, c‐myc, and survivin are among the oncogenes and growth factors that have been demonstrated to be upregulated with activation of the PI3K/AKT/mTOR signaling pathway. One cytokine that tumor cells release is called VEGF, and it plays a key role in angiogenesis, which is crucial for the prognosis and progression of cancer (Mafi et al. 2023) (Figure 1).

In endometrial cancer cells, it has been demonstrated that natural drugs like epigallocatechin gallate (EGCG) decrease the secretion of vascular endothelial growth factor A (VEGFA). This, in turn, inhibits the PI3K/AKT/mTOR/hypoxia inducible factor‐1 (HIF‐1) pathway and suppresses cancer angiogenesis (Gao et al. 2015). Based on these data, it seems sensible to assume that blocking PI3K/AKT signaling results in hyperactivation of the mTOR pathway, which encourages cell division and triggers the cell cycle transition in some cells (Laplante and Sabatini 2013). This eventually develops into a cancer prognosis, according to many publications on human cancer (Murugan 2019). Some typical causes of mTOR hyperactivation include loss of function due to mutations in tumor suppressors such as neurofibromin 1/2 (NF1/2), oncogenic mutations in KRAS, PIK3CA, phosphatase and tensin homolog (PTEN), AKT, or tuberous sclerosis 1/2 (TSC1/2) (Grabiner et al. 2014). Moreover, there is a strong correlation between the activation of the PI3K/AKT/mTOR pathway and the acquisition of Epithelial‐Mesenchymal Transition (EMT) in cancer cells (Guo et al. 2016; Chang et al. 2013). The PI3K/AKT/mTOR signaling pathway may also affect cancer cells and host immunity.

## 2 Mechanism of mTOR Regulation in Cancer Pathways

### 2.1 Autophagy

Autophagy, which plays crucial roles in cellular homeostasis and cell survival in both healthy and pathological conditions, is the product of the evolution of the conserved catabolic process (Boya et al. 2013). According to White (2015) and Kimmelman and White (2017), it is also thought of as a survival‐promoting route that regulates cancer cells and has a complicated role in the growth, development, and propagation of cancer cells. Reactive oxygen species (ROS) buildup and hunger trigger the induction of autophagy (Yun et al. 2020). Two main complexes trigger autophagy: the Unc‐51‐like kinase 1 (ULK‐1) complex and the class 3 phosphoinositide 3‐kinase (PI3K) complex, which comprises Beclin‐1 (Birgisdottir et al. 2019). Activation of the PI3K/AKT/mTOR signaling pathway inhibits it. Growth hormones, amino acids, and ROS may all promote the inhibition of autophagy by activating protein kinases such as mTOR (Lamb et al. 2013; Russell et al. 2014). Moreover, class 1 PI3K diminishes autophagic flow by activating the mammalian rapamycin (mTOR) target, which lowers ULK‐1 (Kim et al. 2011).

The process of macroautophagy results in the emergence of double‐membrane‐bound vesicles called autophagosomes. Transforming a cytosolic truncated protein (LC3‐I) into a phosphatidylethanolamine‐conjugated variant (LC3‐II) results in the formation of an autophagosome. When a lysosome and an autophagosome combine, an autolysosome is created. The autophagic cargo in the autolysosome destroys the autophagy substrates LC3B‐II and p62 (ubiquitin binding protein). The formation of p62 and LC3B‐II aggregates is thought to be a trustworthy sign of a reduction in autophagic flux. Recently, autophagy and TLR signaling were connected (Into et al. 2012). TLR4 inhibition has the potential to decrease cell proliferation; nevertheless, the autophagy‐induced survival mechanism is a conserved catabolic system that has evolved over time and is engaged in a variety of physiological processes, including host protection, cellular metabolism, and longevity (Kuballa et al. 2012; Tey and Khanna 2012).

Most nanoparticles have been shown to aggregate in acidic vesicular organelles such as endosomes and lysosomes, and lysosomes play a crucial role in amino acid‐induced mTORC1 activation (Nel et al. 2009; Parveen et al. 2012; Lunova et al. 2019). Because mTOR signaling is modulated, targeting cancer cell lysosomes with nanoparticles may be a practical strategy for chemotherapeutic treatment. It could be possible to improve the curative benefits of selective mTOR inhibitors by combining them with nanoparticles. Moreover, modifying the surface of nanomaterials could impact mTOR signaling in a unique way. For example, polystyrene nanoparticles with carboxyl groups stimulate mTOR, while those with amino groups inhibit mTOR signaling.

**FIGURE 2:**
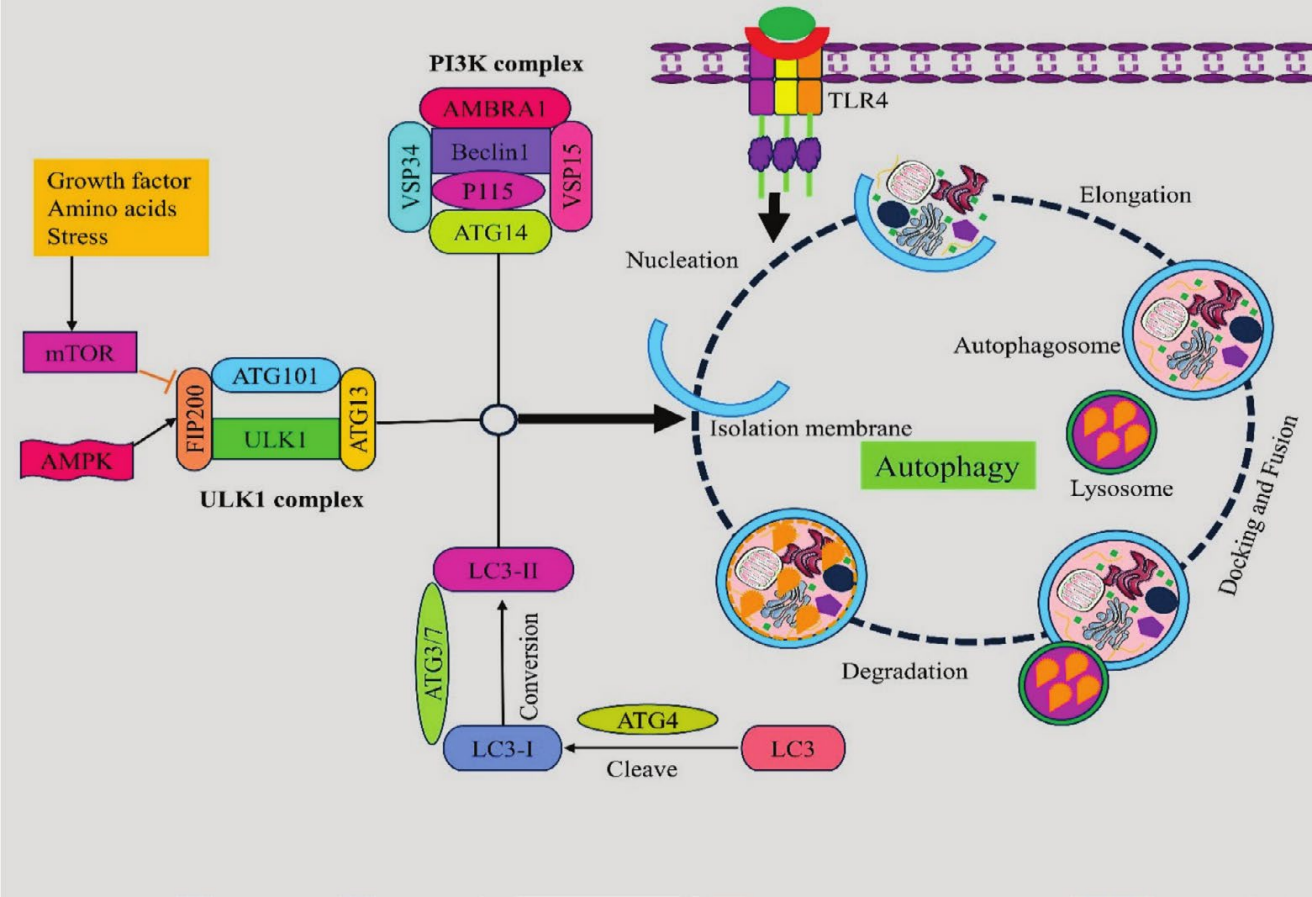
The autophagic mechanisms by regulating mTOR signaling pathways in oncological disorders.

Research on the molecular mechanisms underlying the modification of mTOR signaling by nanoparticles is necessary to maintain the targeted therapeutic effect on cancers. Moreover, as the lysosome is the site of both mTORC1 activation and nanoparticle accumulation, more research is required to understand the relationship between lysosome‐mTORC1 and nanoparticles in cancer cells. Research on the use of mTOR inhibitors in conjunction with tailored nanoparticles to treat certain cancers is essential (Allemailem et al. 2021). The autophagic mechanisms by regulating mTOR signaling pathways in various cancers are displayed in Figure 2.

### 2.2 Apoptosis

**FIGURE 3:**
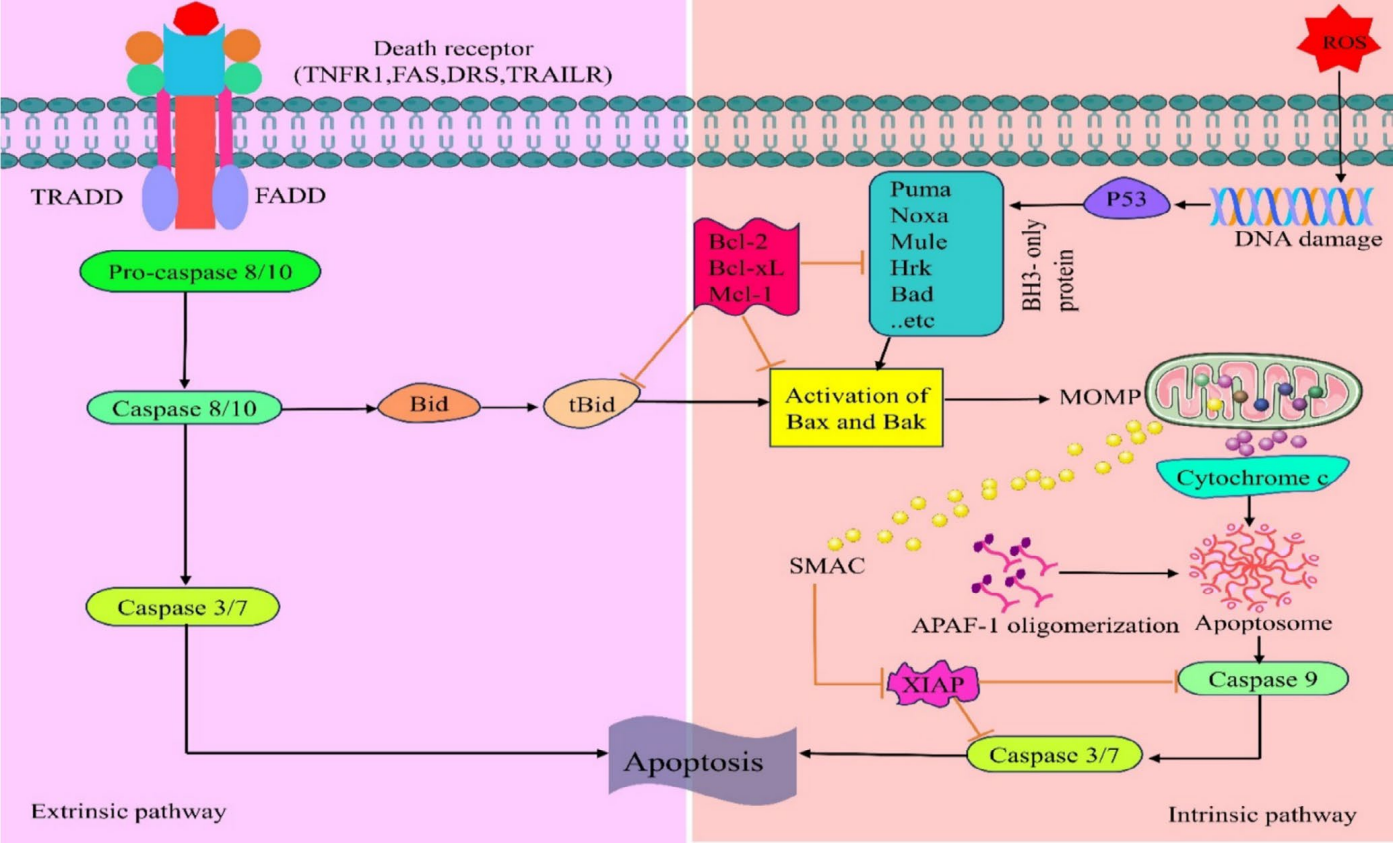
The mechanism of apoptosis by regulating mTOR signaling pathways in oncological disorders.

Apoptosis, which is triggered by many anticancer medications, is another crucial mechanism in the therapy of cancer (Pistritto et al. 2016). Two major molecular pathways are involved: extrinsic (mediated by death receptors) and intrinsic (mitochondrial) (Putcha et al. 2002; Elmore 2007). The Bcl‐2 and caspase families are the main groups affected by the intrinsic pathway (Putcha et al. 2002). TNF activates caspase‐8 by interacting with TNF receptor 1 (TNFR1) and cleaving procaspase‐3 into its active form (Elmore 2007). By activating caspases through the mitochondrial pathway or releasing cytochrome C, phytochemicals can induce apoptosis. An apoptosis‐inducing factor (AIF) is produced as a result of them (Jung et al. 2007; Fernández‐Lázaro et al. 2024). It has been demonstrated that several of these compounds suppress anti‐apoptotic factors while increasing the production of pro‐apoptotic proteins such as caspase‐3 and BAX (Wang et al. 2015). Figure 3 depicts the apoptotic mechanism in tumors that is mediated by mTOR signaling pathways.

### 2.3 Inhibition of Proliferation

Cancer cells change their metabolism to have access to nutrients, which helps them grow and proliferate. Pathways that promote food absorption control have a similar alteration in cancers, notwithstanding the variety of genetic alterations that lead to carcinogenesis (Pazhooh et al. 2021). Therefore, restricting the intake of nutrients and other essential materials (such growth hormones) can prevent the development and metabolism of malignant cells, making this a potentially effective therapeutic approach for the treatment of cancer (Feitelson et al. 2015; Zhu and Thompson 2019). According to Mayer and Arteaga (2016), hyperactivation of mTOR signaling promotes cell proliferation and metabolism, both of which are involved in the genesis and growth of tumors. According to Watanabe et al. (2011), the signaling pathway is mainly involved in cell growth, survival, metabolism, production of proteins, and homeostasis. Through SGK1 kinase, the mTOR signaling system synchronizes anabolic processes with energy, oxygen, and food availability as well as external stimuli to control cell growth and proliferation (Dibble and Manning 2013; Mori et al. 2014). Consequently, natural mTOR signaling system inhibitors can decrease the growth of malignant cells, making this a prominent therapeutic target in the management of cancer.

## 3 Effect of Plant‐Derived Bioactives on mTOR Pathway

Plant bioactive substances are second‐generation metabolites that have pharmacological or toxicological effects on both humans and animals. These plant‐derived bioactives interact with the mTOR pathway through various mechanisms, modulating subsequent cellular processes. Plant bioactive compounds have the potential to significantly impact the mTOR pathway, offering promising treatment approaches for disorders characterized by mTOR dysregulation (Fakhri et al. 2022). Several plant bioactive compounds have been identified to affect the mTOR pathway. The widely recognized plant bioactive ingredient resveratrol, which is present in grapes and red wine, stimulates AMP‐activated protein kinase (AMPK), a cellular energy gauge. The tuberous sclerosis complex (TSC) proteins are phosphorylated and activated during AMPK activation, hence inhibiting mTOR function and suppressing the mTOR pathway. Resveratrol may have curative properties because of its activation of AMPK and reduction of mTOR signaling, which reduce protein synthesis and promote autophagy (Brockmueller et al. 2021). Curcumin, a bioactive component of turmeric, has an effect on the mTOR pathway as well. It controls the production of microRNAs linked to mTOR signaling and inhibits the PI3K/AKT pathway, a major upstream activator of mTORC1. Furthermore, curcumin modifies mTOR activity by activating AMPK. By inhibiting mTOR‐mediated processes, curcumin exhibits potential anti‐inflammatory and anticancer effects (Khan et al. 2020). EGCG, a green tea catechin, has been demonstrated to reduce mTOR signaling by activating AMPK and decreasing AKT, a prominent mTORC1 activator. This results in decreased cell proliferation and the activation of autophagy. EGCG's mTOR inhibitory actions are linked to its anti‐cancer chemo‐preventive properties (Seok et al. 2019). Berberine, an isoquinoline alkaloid found in plants like Goldenseal and barberry, inhibits mTORC1 activation through the AMPK‐dependent pathway, thereby suppressing mTOR signaling. Berberine's potential therapeutic benefits against various conditions such as cancer, diabetes, and cardiovascular diseases may be influenced by its ability to modulate the mTOR pathway (Rauf et al. 2021). Triterpenoids such as celastrol and ursolic acid also impact the mTOR pathway. Celastrol, derived from *Tripterygium wilfordii*, inhibits mTOR signaling by reducing mTORC1 activity and inducing autophagy. Ursolic acid, found in plants like rosemary and apple peels, inhibits mTORC1 signaling by reducing the phosphorylation of downstream targets. These compounds have implications for cancer prevention and treatment (Gill et al. 2016). Sulforaphane, derived from cruciferous vegetables like broccoli and cauliflower, has demonstrated mTOR inhibitory effects and is associated with the mechanisms involved in reduced cancer cell proliferation (Xie et al. 2022). Table 1 shows different bioactive compounds extracted from various plants to target pathways regulating cancers.

**TABLE 1:** Plant‐derived bioactive compounds acting through mTOR pathways to treat various oncological disorders.

Plant bioactive compounds	Plant source	Target pathway	Type of cancer	References
Resveratrol	Grapes, red wine	AMPK, tuberous sclerosis complex (TSC) proteins	Breast cancer, prostate cancer, colorectal cancer	Alayev et al. (2015)
Curcumin	Turmeric	Phosphoinositide 3‐kinase (PI3K)/AKT pathway, AMPK	Breast, colorectal, pancreatic	Hamzehzadeh et al. (2018)
Epigallocatechin gallate (EGCG)	Green tea	AMPK, AKT	Breast, prostate, lung	Singh et al. (2011)
Quercetin	Onions, apples, berries, tea	PI3K/AKT pathway, mTORC1	Lung, breast, colon	Mirza‐Aghazadeh‐Attari et al. (2020)
Caffeic acid	Coffee, fruits, vegetables	PI3K/AKT pathway, mTORC1	Liver, colon, breast	Narayanankutty (2021)
Ursolic acid	Apple peels, rosemary	mTORC1 downstream targets (S6 kinase, 4E‐BP1)	Breast, colorectal, prostate	Zhao et al. (2020)
Berberine	Goldenseal, barberry	AMPK, mTORC1	Breast, gastric liver, colorectal, pancreatic	Zhu et al. (2022)
Celastrol	*Tripterygium wilfordii* (Thunder God Vine)	mTORC1, autophagy	Lung, pancreatic, breast, colon, leukemia	Haroon and Kang (2020)
Kaempferol	Chinese medicinal plants, herbs, fruits, beans	mTORC1, mTOR‐related gene expression	Lung, liver, colon	Imran et al. (2019)
Genistein	Soybeans, legumes	PI3K/AKT pathway, mTORC1	Breast, prostate, ovarian	Sharifi‐Rad et al. (2021)
Fisetin	Strawberries, apples, onions	PI3K/AKT pathway, mTORC1	Breast, prostate, ovarian	Rengarajan and Yaacob (2016)
Pterostilbene	Blueberries, grapes	PI3K/AKT pathway, mTORC1	Breast, lung, liver, prostate, colorectal	Levenson and Kumar (2020)
Olive leaves	Olive leaves (*Olea europaea L*.)	PI3K/AKT pathway, mTORC1	Breast, lung, colorectal, prostate	Albogami and Hassan (2021)
Sulforaphane	Cruciferous vegetables (broccoli, cauliflower)	AMPK, mTORC1	Breast, bone, lung, colorectal, bladder, prostate	Esteve (2020)

## 4 Effect of Plant Bioactives Against mTOR Pathways Mediated Oncological Disorders

### 4.1 Oral Cancer

The anticancer properties of lycopene (LP) on oral cancer (OC) cells indicate that it may stimulate the PI3K/AKT/mTOR signaling pathway, which would suppress EMT and boost OC cell death. Wang et al. (2020) found that the anti‐cancer properties of LP were linked to a dose‐related rise in apoptosis and a reduction in the growth, building of colonies, movement, and penetration of OC cells. To understand the mechanism underlying the regulatory effect of LP on OC, western blot was used to analyze the EMT markers (E‐cadherin and N‐cadherin) and important proteins associated with the PI3K/AKT/mTOR signaling pathway. In OC cells, LP treatment increased the ratio of EMT markers while decreasing the expression ratios of p‐PI3K/PI3K, p‐AKT/AKT, and p‐mTOR/mTOR. Cinnamomum zeylanicum extract (CZE) and its active component cinnamaldehyde (CIN) have demonstrated anti‐tumor properties against oral cancer. Laboratory studies indicated that both CZE and CIN effectively reduced the growth and proliferation of oral cancer cells, triggered apoptosis, and promoted autophagy. Additionally, they were found to inhibit the invasion and movement of NF‐κB into the cytoplasm, while also lowering the expression of several pathways related to PI3k‐AKT–mTOR (Aggarwal et al. 2022). Artonin E (AA2) and artobiloxanthone (AA3) induce apoptosis in oral cancer cells because they activate both caspase‐3 and caspase‐9, integral proteins in apoptosis. Both inhibited the expression of oncogenic proteins Bcl‐2, COX‐2, VEGF, and MMP‐9 connected with the survival of tumors, inflammatory, angiogenetic, and metastatic diseases. Moreover, AA2 and AA3 affect the Akt/mTOR and STAT‐3 signaling pathways by inhibiting the key cellular processes of cell proliferation, survival, and tumor growth (Aswathy et al. 2024).

### 4.2 Breast Cancer

It is well known that epidermal growth factor (EGF) phosphorylates mTOR and the associated mitogen‐activated protein kinase, which subsequently synthesizes genes linked to apoptosis, including hypoxia‐triggered factor 1‐alpha (HIF1). Jiang and Liu (2008) reported that the activation of the mTOR‐mediated HIF1 factor enhances several activities of endothelial and cancer cells, including the production of proteins, blood vessel development, the spread of cancer cells, emigration, division, and the death of cells. Park et al. (2016) investigated this pathway and found that pomolic acid prevents mTOR from being phosphorylated by EGF. HIF1 is also an important modulator of VEGF synthesis and a critical regulatory protein downstream of mTOR signaling. Tsai and Wu (2012). Increased HIF activation, which controls carcinogenesis, angiogenesis, and tumor expansion via VEGF, is generally associated with increased mTOR stimulation of following proteins, such as 70S6K and 4E‐BP1 (Guru et al. 2015). It has been found that pomolic acid inhibits the phosphorylation of mTOR and subsequent proteins, such as p70S6K and 4E‐BP1, in cells of breast carcinoma, which is triggered by EGF.

Muhammad et al. (2017) found that bitter melon ( *Momordica charantia* ) extract (BME) blocks mTOR/AKT signaling, which in turn promotes autophagy. Several investigations have demonstrated the therapeutic potential of mTOR kinase in the management of cancer. Moreover, BME stimulated AMPK in breast cancer cells, which controls cellular metabolism in an ATP/AMP ratio‐dependent approach to assist in maintaining a proper equilibrium for energy (Mihaylova and Shaw 2011).

Khan et al. (2021) investigated if the activity of important indicators, such as the tumor suppressor p53, pro‐apoptotic Bax, anti‐apoptotic Bcl‐2, and effector caspase‐3, increased in response to ethanolic Ajwa Dates Pulp Extract (ADPE). To gain more insight into the intracellular signaling system, the regulation of mTOR and AKT molecules was also investigated. After 48 h of ADPE treatment, the MDA‐MB‐231 cancer cell line underwent apoptosis as a result of decreased p‐AKT and p‐mTOR levels. According to these results, ADPE inhibits the AKT/mTOR signaling pathway, which is important for controlling the development and division of breast cancer cells.

It has been discovered that the effects of ursolic acid (UA) on cell survivability and mortality are modulated via the PI3K/AKT and MAPK cellular signaling pathways (Achiwa et al. 2007). Two higher dosages of UA (0.10% and 0.25%) were investigated by De Angel et al. (2010) on MMTV‐Wnt‐1 breast tumors. The results showed a significant reduction in ribosomal protein S6 (Ser265/569), phosphorylation of AKT, and MAPK cellular signaling. These findings validate UA's capacity to alter the AKT/mTOR pathway, inducing apoptosis and so impeding the growth of tumors. Comparably, it has been demonstrated that flavonoids and isoflavones extracted from *Tephroseris kirilowii* and Astragalus membranaceus cause cell death in various breast cancer in humans cell types by obstructing the PI3K/AKT/mTOR pathway (Zhang et al. 2018; Zhou et al. 2018). It has been shown that extracts of rosemary ( *Rosmarinus officinalis* ) reduce AKT and mTOR phosphorylation/activation, suggesting potential anticancer benefits (Jaglanian and Tsiani 2020). Resveratrol effectively suppressed the growth of breast cancer cells by causing developmental suspensions through the disruption of mTORC1 signaling networks and targeting AKT, as reported by Alayev et al. (2017).

Ganesan et al. (2024) evaluated the role of ononin, an isoflavonoid, on lung metastasis in triple‐negative breast cancer, showing very encouraging results. Ononin resulted in 50% inhibition of the proliferation of TNBC cells and decreased colony formation by 60% as measured in colony‐forming assays. The invasion and motility of TNBC cells decreased by 40% as determined using Transwell and wound healing assays. Ononin also resulted in a 45% reduction in tumor growth in TNBC xenograft models and decreased lung metastasis by 50%. It further reversed epithelial‐mesenchymal transition (EMT), downregulated EMT markers, and matrix metalloproteinases by 30% as assessed by western blotting. It also inhibited EGFR phosphorylation by 40%, which resulted in the inhibition of PI3K/Akt/mTOR signaling pathways with a 35% reduction. It was nontoxic to the liver and kidneys; thus, it is not toxic to the body system, and these findings make ononin a therapeutic agent for targeting the pathways mediated by EGFR in the metastasis of TNBC.

### 4.3 Lung Cancer

The transcription levels of AKT and mTOR mRNA in cells were assessed by Liu, Gao, et al. (2018) after curcumin therapy. They identified a substantial decrease in the transcription levels of AKT and mTOR mRNA in the treated sample as contrasted to the standard. The researchers used mTOR and PI3K/AKT inhibitors, such as rapamycin and LY294002, to evaluate curcumin in order to validate its inhibitory effect on these pathways. Key signaling molecules like mTOR, p‐mTOR, AKT, and p‐AKT were expressed in comparison to the reference cell line using western blotting, as mRNA does not accurately reflect protein expression. Curcumin therapy, in conjunction with prior treatment with rapamycin and LY294002, enhanced starvation and death in cancer cells by inhibiting mTOR signaling. Ahmad et al. (2023) explored the anticancer properties of two plant‐derived bioactive compounds, curcumin and plumbagin, focusing on their impact on the PI3K/AKT/mTOR pathway. Molecular docking analysis revealed that the combination of curcumin and plumbagin was more effective in inhibiting this pathway than either compound alone. In particular, the binding free energy analysis indicated that the complexes formed by PI3K, AKT, and mTOR with the combination of curcumin and plumbagin exhibited significantly higher binding free energy levels, suggesting a more effective synergistic approach to targeting the pathway.

### 4.4 Leukemia

Using gene expression and protein immunoblotting assays, Tlili et al. (2021) examined the impact of *Rhus tripartita* extracts on the PI3K/AKT/mTOR signaling pathways in acute monocytic leukemia THP‐1 cells. The findings suggest that *R. tripartita* extracts induce cell cycle arrest and apoptosis via the PI3K/AKT/mTOR signaling pathway by reducing phosphorylation of mTORC1 substrate S6Ser2040‐244 and mTORC2 downstream target AKT‐Ser473 in acute monocytic leukemia THP‐1 cells. Moreover, it is known that gallic acid inhibits AKT/mTOR signaling, which leads to mitochondrial dysfunction, an energy crisis, and the death of acute myeloid leukemia cells (Gu et al. 2018). Targeting key components of tumor suppressors, such as phosphatase and tensin homolog (PTEN), tuberous sclerosis 1/2 (TSC1/2), neurofibromin 1/2 (NF1/2), or oncogenic mutations in KRAS, PIK3CA, or AKT, may represent an effective treatment to kill acute myeloid leukemia and leukemia stem cells, according to growing evidence (Grabiner et al. 2014). Al‐Rawashde et al. (2022) examined the potential anti‐leukemic properties of thymoquinone (TQ), a compound derived from *Nigella sativa* , specifically on acute myeloid leukemia (AML) and chronic myeloid leukemia (CML) cells by targeting the JAK/STAT and PI3K/Akt/mTOR signaling pathways. Treatment with TQ in FLT3‐ITD positive MV4‐11 AML cells and BCR‐ABL positive K562 CML cells led to a dose‐ and time‐dependent reduction in cell proliferation, significant downregulation of PI3K, Akt, and mTOR, and an increase in PTEN expression at both mRNA and protein levels. TQ effectively inhibited both the JAK/STAT and PI3K/Akt/mTOR pathways, resulting in a decrease in leukemia cell proliferation. These results indicate that TQ may be a promising therapeutic option for AML and CML, highlighting the need for further in vivo studies and clinical trials to assess its molecular mechanisms and effectiveness.

### 4.5 Gastric Cancer

It has been demonstrated that Thymoquinone, a bioactive lactone produced by *N. sativa* seed oil (black cumin), stimulates death in gastric cancer cells through disrupting the PI3K/AKT/mTOR signaling system. The treatment of thymoquinone induced a dose‐dependent decline in the concentrations of total AKT, mTOR1, mTOR2, and pI3K, but not in the levels of p‐AKT (S473), p‐AKT (T308), p‐mTOR1, p‐mTOR2, or pPI3K. Moreover, thymoquinone suppressed the production of mesenchymal genes such as N‐cadherin, Vimentin, and TWIST and hindered the spreading of gastric cancer cells. However, in gastric cancer cells given thymoquinone, there was a substantial increase in the regulation of epithelial marks like cytokeratin‐19 and E‐cadherin. Wu et al. (2024) investigated the therapeutic effects and mechanisms of gypenoside, a natural extract from *Gynostemma pentaphyllum*, on gastric cancer. Gypenoside was found to induce apoptosis in HGC‐27 and SGC‐7901 gastric cancer cells in a dose‐ and time‐dependent manner by targeting the PI3K/AKT/mTOR signaling pathway, as confirmed through network pharmacology, molecular docking, and western blot analyses. Mechanistically, gypenoside inhibited the phosphorylation of STAT3, which led to a reduction in PD‐L1 transcription in gastric cancer cells. This inhibition enhanced the antitumor activity of CD8+ T cells, as shown in coculture experiments. In vivo studies further supported gypenoside's antitumor effects, revealing significantly downregulated PD‐L1 expression in tumors treated with gypenoside. These findings suggest that gypenoside induces apoptosis through PI3K/AKT/mTOR inhibition and boosts T‐cell‐mediated immunity by suppressing STAT3 phosphorylation and PD‐L1 expression, making it a promising candidate for gastric cancer immunotherapy.

### 4.6 Hepatic Cancer

Dihydromyricetin, a flavonoid compound derived from Ampelopsis grossedentata, lowers mTOR by altering its precursor signaling channels, according to Xia et al. (2014) in an additional study. The western blot approach was used to investigate the class III phosphatidylinositol 3‐kinase/phosphoinositide‐dependent protein kinase 1/protein kinase B (PI3K/PDK 1/AKT) and extracellular signal‐regulated kinase (AMPK) pathways, which are mTOR‐related upstream signaling molecules. These processes have been connected to the hepatic cancer (HepG2) cells' activation of autophagy. Dihydromyricetin treatment increased PI3K expression while decreasing AKT and ERK1/2 phosphorylation. One master kinase that might hinder autophagy is PDK 1. Furthermore, by reducing the induction of AKT as well as subsequent targets of mTOR, cannabisin B, a naturally occurring substance isolated from the hempseed ( *Cannabis sativa* L.) hull, restricts cell growth and leads to cell death in human (HepG2) hepatocarcinoma cells (Chen et al. 2013). Prunetrin (PUR), which is derived from *Prunus* species, shows anti‐cancer properties in Hep3B liver cancer cells. It halts the cell cycle at the G2/M phase by lowering the levels of Cyclin B1, CDK1/CDC2, and CDC25c, and it triggers intrinsic apoptosis via the mitochondrial pathway. This is supported by the increased presence of cleaved caspase‐3, caspase‐9, PARP, and Bak, along with a reduction in Bcl‐xL levels. PUR also inhibits the AKT/mTOR signaling pathway and activates p38‐MAPK in a dose‐dependent manner, leading to reduced cell proliferation and increased apoptosis. These results indicate that PUR could be a promising therapeutic agent for hepatocellular carcinoma (Abusaliya et al. 2023).

### 4.7 Cervical Cancer

A flavonoid called licochalcone A (LiclA), which was isolated from the Batalin root of Glycyrrhiza inflata, inhibits the PI3K/AKT/mTOR signaling pathway in human cervical carcinoma lineages, triggering autophagy and cell death. LiclA decreased the expression of phosphor‐mTOR (ser2448), phosphor‐mTOR (ser2481), Raptor, and Rictor in a dose‐ and time‐dependent manner, according to Tsai et al. (2015). Moreover, rapamycin, a mTOR inhibitor, pretreatment in cancer cells showed a synergistic effect with LiclA in causing death, cancer prevention, and autophagy induction. Chrysotoxine (CTX) has two mechanisms through which it accomplishes an anti‐cervical cancer effect; it is inducing ferroptosis and preventing the PI3K/AKT/mTOR signaling pathway. CTX induces ferroptosis by elevating reactive oxygen species (ROS) and lipid peroxides, causing oxidative bumb. CTX causes this via the p53/GPX4/SLC7A11 axis, in which p53 is activated by CTX and the expression of its inhibitor SLC7A11, and functionally, CTX blocks GPX4, a master regulator of cellular defense against lipid oxidation. Excessive lipid peroxides and ROS drive ferroptotic cell death. Finally, CTX also suppresses the PI3K/AKT/mTOR axis, which is crucial for cell maintenance and survival in addition to phosphatidylinositol metabolism. CTX inhibits cellular metabolic activity and protein synthesis by interfering with this pathway, thus inducing cell death that limits tumor development (Zhou et al. 2024).

### 4.8 Urinary Bladder Cancer

Gartanin, a xanthone that occurs naturally in *Garcinia mangostana* , has shown promise as a tumor suppressor against human urinary bladder cancer. Studies have shown that gartanin therapy inhibits downstream activities of the mTOR process (p70S6 and 4E‐BP1) by two processes including AMPK stimulation as well as AKT suppression, accordingly (Liu et al. 2013). These studies also showed that phosphorylation degrees of mTOR and AMPK in RT4 cells were not significantly changed, yet it led to a dose‐dependent decrease in both phospho‐AKT and overall AKT levels.

Furthermore, Kavalactones, a type of lactone compound found in the roots of the kava plant ( *Piper methysticum* ), consist of docetaxel, yangonin, and flavokawain A. These compounds have a tendency to hinder the mTOR pathway by altering its downstream (rpS6, p70S6K, and 4EBP1) and upstream (LKB1, AKT signaling, and PRAS40) instances. This is compatible with the dropped viability of human bladder cancer cell lines (Liu et al. 2017). By modifying the AKT/mTOR signaling pathway through microRNA regulation, resveratrol promotes autophagy in bladder cancer cells (Zhou et al. 2014). By targeting the PI3K/AKT/mTOR pathway, paclitaxel and curcumin reduce autophagy in the bladder cancer cells (Khan et al. 2020).

Sulforaphane, an isothiocyanate in cruciferous vegetables, has potent anticancer effects mostly against bladder cancer. It causes apoptosis, cell‐cycle arrest, and suppresses bladder cancer cell proliferation, invasion, and metastasis. Sulforaphane also inhibits gluconeogenesis in bladder cancer and enhances chemotherapeutic drug action. It modulates its targeted basal molecular cascades, including the PI3K/AKT/mTOR pathway, MAPK, NfκB ZO‐1, NCAT, Nrf2, and miR‐124/IL‐6R/STAT3, which are major in bladder cell multiplication. It also affects bladder cancer stem cells and thus could be important in overcoming chemoresistance (Zuo et al. 2023).

*G. pentaphyllum*, by its active compound gypenosides, showed strong anticancer activity against bladder cancer. Bioactive ingredients of 10 compounds and 163 gene targets were identified using network pharmacology. VEGFA, STAT3, and PI3KCA were found to be critical candidates. The PI3K/AKT/mTOR signaling pathway is inactivated by the mechanism of action. Molecular docking confirmed the high binding affinity of gypenosides to PI3K, AKT, and mTOR. In vitro, the gypenosides caused the disruption of the signaling pathway through which bladder cancer cells underwent apoptosis, thus bringing about reduced proliferation and colony formation. In vivo studies further corroborated these results as gypenosides greatly inhibited tumor growth (*p* < 0.05), largely by promoting apoptosis through the PI3K/AKT/mTOR pathway suppression (Li et al. 2022).

### 4.9 Colon Cancer

The Ras protein is oncogenically activated in tumor cells, and this can be regulated by chemical compounds to reduce the proliferation of cancer cells. Kumar and Agnihotri (2019) combined only one intraperitoneal 1,2‐dimethylhydrazine (DMH) with a week‐long exposure to the inflammatory agent dextran sulfate sodium (DSS) to synthetically induce colorectal cancer (CRC) in mice. This resembled the unusual crypt foci–adenoma–carcinoma sequence that occurs in human CRC. It has been identified that the Ras oncoprotein connects with PI3K to phosphorylate and stimulate AKT. An alkaloid termed piperlongumine (PL), which is extracted from *Piper longum* Linn, substantially suppressed PI3K levels and Ser473 phosphorylation of AKT, thereby assessing the activity of both AKT/mTORC1 and mTORC2.

Additionally, after receiving DMH, DSS, and PL together, there was a decline in the gene transcription factor of NF‐kB, which has been proposed to promote a defense against apoptosis by enhancing levels of anti‐apoptotic proteins like Bcl‐2. Liu, Zhao, et al. (2018) found that the stimulated portion of clove produces apoptosis in colorectal cancer HCT‐116 cells by a PI3K/AKT/mTOR‐mediated autophagic route. The key component in cloves, oleanonic acid, diminished phospho‐Akt levels, downregulated phospho‐mTOR, and blocked PI3K phosphorylation in a dose‐dependent approach. Its combination activity using the PI3K regulator LY294002 proved more obvious in decreasing the relative amounts of p‐AKT/AKT and p‐mTOR/mTOR.

Atractylodin (ATD) suppresses mTOR and p38MAPK phosphorylation and elevates Beclin‐1 expression to stimulate autophagy in a cholangiocarcinoma cell line (Acharya et al. 2021). *Atractylodes lancea* is the principal origin of ATD. The influence of ATD on PI3K phosphorylation was dose‐dependent. According to Corti et al. (2019), AKT is a PI3K subsequent regulator protein that modulates angiogenesis, cell growth and multiplication alongside controlling the activity of the downstream mTOR protein. By inhibiting AKT, which either actively phosphorylates mTOR or slowly lowers the tuberous sclerosis complex, a negative regulator of mTOR, ATD substantially enhanced autophagy. Moreover, relative to the untreated control group, as the amount of ATD increased, so did the level of total mTOR and p‐mTOR.

In human colon tumor cells HCT116 and HT29, the curative impacts of curcumin combined with 5‐Fluorouracil (5‐Fu) on cellular autophagy have been investigated (Zhang et al. 2017). In order to discover the molecular process that activates the autophagic reaction, this research explored alterations produced to the fundamental autophagic machinery, particularly ULK1 and its upstream triggers AKT, mTOR, and AMPK. The findings suggested that mono‐5‐Fu, without altering AMPK signaling, regulated P‐ULK1(Ser317) and suppressed AKT/mTOR phosphorylation, causing autophagy in HCT116 and HT29 cells. Nevertheless, it was found that the combined administration of curcumin and 5‐Fu reduced all rates of serine/threonine kinases, including P‐AKT, P‐mTOR expressions, P‐AMPK, and P‐ULK1(S317). El‐Wetidy et al. (2024) investigated the apoptotic pathway by which Urolithin A (UA) exerts its inhibition of colorectal cancer (CRC) cells. Treatment with UA of HT29 cells was associated with apoptosis and suppressors of death, such as U0126 and LY294002, markedly protected the cells from death. Conversely, these suppressors showed little inhibition toward metastatic SW620 cells and indicated that the p‐AKT/PI3K/mTOR pathway was involved in CRC. Western blotting confirmed that the effects of UA on p‐AKT/PI3K/mTOR signaling are related to its effect on increasing the phosphorylation of ERK and AMPK in HT29 cells. UA lowered p‐AKT and mTOR but enhanced p‐c‐RAF and PTEN in SW620 cells, thus proposing a defense mechanism against apoptosis induced by UA. All these results illustrate that UA is potentially able to modulate the pathways of CRC progression and metastasis.

## 5 Monitoring of mTOR Signaling

Evaluating mTOR activity is essential to comprehending the intricate signaling network governing cell division, development, and metabolism. Numerous physiological and pathological processes, such as cancer, metabolic issues, and neurological illnesses, depend on the mTOR pathway. Scientists have come to understand that tracking mTOR activity is essential for gaining understanding of the underlying causes of illness and developing tailored treatments (Swiech et al. 2008; Yu et al. 2011).

Since 2000, scientists have been attempting to create trustworthy stand‐in indicators to gauge how well mTOR inhibitors are performing as cancer treatments. But issues with biomarker repeatability and variability have impeded advancement. The impact of mTOR inhibitors on molecular markers such as p‐S6K1 and p‐4EBP1 in different tissues, including skin, peripheral blood mononuclear cells (PBMCs), and tumor biopsies, has been examined in a number of studies. Regretfully, these studies have shown inconsistent and preliminary results. Furthermore, the effect of mTOR inhibitors on tumor metabolism and angiogenesis has been evaluated using imaging techniques such as positron emission tomography (PET) scans. PET imaging can reveal information about mTOR activity and the response to therapy by identifying the absorption of 18F‐fluorodeoxyglucose (FDG), a marker of tumor metabolism influenced by mTOR signaling. However, more study is needed to establish a definitive relationship between mTOR imaging and clinical results (Watanabe et al. 2011).

To overcome the obstacles associated with evaluating mTOR inhibitors, continuing research focuses on establishing accurate and dependable methodologies. In recent years, liquid biopsy has developed as a viable non‐invasive technique for tumor identification and surveillance, providing vital information regarding tumor features and dynamics. By analyzing circulating tumor DNA (ctDNA), circulating tumor cells (CTCs), and circulating non‐coding RNAs (ncRNAs) extracted from diverse bodily fluids, liquid biopsy can uncover medication resistance, evaluate therapeutic response, and diagnose cancer early (Moldogazieva et al. 2021; Gao et al. 2021). In addition to liquid biopsy, biosensing technology has gained significance in monitoring mTOR signaling. The development of biosensors and genetically encoded reporters enables real‐time monitoring of mTOR signaling dynamics within living cells and animal models. These biosensors detect and quantify mTOR activity using techniques such as fluorescence resonance energy transfer (FRET), bioluminescence, or other signaling processes. Researchers can track mTOR signaling in response to different stimuli, therapeutic agents, or genetic manipulations by incorporating these biosensors into cancer cell lines or tumor models, providing valuable insights into its role in tumor growth and treatment resistance (Bouquier et al. 2020; Lin et al. 2019; Mehta and Zhang 2021; Zhou et al. 2015).

## 6 Conclusion and Future Scope

Studying the modulation of the mTOR signaling system in vivo by inducing autophagy with bioactive chemicals and monitoring mTOR activity could be a focus of future cancer treatment research. These studies offer promise as advanced approaches for dealing with various tumor responses in the human body. Although it has been shown that plant extracts can induce apoptosis in cancer cells, the precise molecular processes at play—most notably, the role of the PI3K/AKT/mTOR signaling pathway—remain unclear. Further research with PI3K/AKT inhibitors or siRNA trials is required to demonstrate a link between plant bioactive compounds and the mTOR pathway, offering light on their potential function in cancer treatment.

A thorough investigation of the molecular links between bioactive substances, mTOR signaling, and autophagy activation can pave the way for the development of customized medications with improved efficacy and fewer side effects. Monitoring strategies for mTOR activity must be used during this research. Real‐time analysis of bioactive substance regulation of mTOR signaling provides vital insights into the dynamic changes occurring within the system. For exact quantification and visualization of mTOR activity in response to treatment, molecular probes, imaging tools, and novel approaches can be used.

By unraveling the intricate molecular interactions between bioactive compounds, the mTOR signaling pathway, autophagy activation, and monitoring mTOR activity, our understanding of cancer biology can significantly advance. These discoveries serve as a solid foundation for refining therapeutic approaches that effectively address distinct tumor responses. The goal is to develop targeted and customized medicines with improved efficacy and minimal side effects, revolutionizing cancer treatment strategies.

## Author Contributions

**Sonali Khanal:** investigation, methodology, writing – original draft. **Manjusha Pillai:** investigation, methodology, writing – original draft. **Md. Shimul Bhuia:** writing – review and editing. **Muhammad Torequl Islam:** writing – review and editing. **Onkar Sharma:** investigation, methodology, writing – original draft. **Rachna Verma:** conceptualization, supervision, writing – review and editing. **Eugenie Nepovimova:** supervision, writing – review and editing. **Kamil Kuca:** conceptualization, funding acquisition, supervision, writing – review and editing. **Dinesh Kumar:** supervision, writing – review and editing. **Raihan Chowdhury:** supervision, writing – review and editing.

## Conflicts of Interest

The authors declare no conflicts of interest.

## Supporting information


**Data S1.** Supporting Information.

## Data Availability

The authors have nothing to report.

## References

[ptr70051-bib-0001] Abusaliya, A. , S. H. Jeong , P. B. Bhosale , et al. 2023. “Mechanistic Action of Cell Cycle Arrest and Intrinsic Apoptosis via Inhibiting Akt/mTOR and Activation of p38‐MAPK Signaling Pathways in Hep3B Liver Cancer Cells by Prunetrin – A Flavonoid With Therapeutic Potential.” Nutrients 15, no. 15: 3407. 10.3390/nu15153407. 37571343 PMC10420889

[ptr70051-bib-0002] Acharya, B. , W. Chaijaroenkul , and K. Na‐Bangchang . 2021. “Atractylodin Inhibited the Migration and Induced Autophagy in Cholangiocarcinoma Cells via PI3K/AKT/mTOR and p38MAPK Signaling Pathways.” Journal of Pharmacy and Pharmacology 73, no. 9: 1191–1200. 33885818 10.1093/jpp/rgab036

[ptr70051-bib-0003] Achiwa, Y. , K. Hasegawa , and Y. Udagawa . 2007. “Regulation of the Phosphatidylinositol 3‐Kinase‐Akt and the Mitogen‐Activated Protein Kinase Pathways by Ursolic Acid in Human Endometrial Cancer Cells.” Bioscience, Biotechnology, and Biochemistry 71, no. 1: 31–37. 17213663 10.1271/bbb.60288

[ptr70051-bib-0004] Aggarwal, S. , K. Bhadana , B. Singh , et al. 2022. “ *Cinnamomum zeylanicum* Extract and Its Bioactive Component Cinnamaldehyde Show Anti‐Tumor Effects via Inhibition of Multiple Cellular Pathways.” Frontiers in Pharmacology 13: 918479. 10.3389/fphar.2022.918479. 35774603 PMC9237655

[ptr70051-bib-0005] Ahmad, I. , M. Hoque , S. S. M. Alam , T. A. Zughaibi , and S. Tabrez . 2023. “Curcumin and Plumbagin Synergistically Target the PI3K/AKT/MTOR Pathway: A Prospective Role in Cancer Treatment.” International Journal of Molecular Sciences 24, no. 7: 6651. 10.3390/ijms24076651. 37047624 PMC10095292

[ptr70051-bib-0006] Alayev, A. , S. M. Berger , and M. K. Holz . 2015. “Resveratrol as a Novel Treatment for Diseases With mTOR Pathway Hyperactivation.” Annals of the New York Academy of Sciences 1348, no. 1: 116–123. 10.1111/nyas.12829. 26200935

[ptr70051-bib-0007] Alayev, A. , R. S. Salamon , N. S. Schwartz , A. Y. Berman , S. L. Wiener , and M. K. Holz . 2017. “Combination of Rapamycin and Resveratrol for Treatment of Bladder Cancer.” Journal of Cellular Physiology 232, no. 2: 436–446. 27225870 10.1002/jcp.25443

[ptr70051-bib-0008] Albogami, S. , and A. Hassan . 2021. “Assessment of the Efficacy of Olive Leaf (*Olea europaea* L.) Extracts in the Treatment of Colorectal Cancer and Prostate Cancer Using In Vitro Cell Models.” Molecules 26, no. 13: 4069. 10.3390/molecules26134069. 34279409 PMC8272070

[ptr70051-bib-0009] Allemailem, K. S. , A. Almatroudi , F. Alrumaihi , et al. 2021. “Novel Approaches of Dysregulating Lysosome Functions in Cancer Cells by Specific Drugs and Its Nanoformulations: A Smart Approach of Modern Therapeutics.” International Journal of Nanomedicine 16: 5065–5098. 10.2147/ijn.s321343. 34345172 PMC8324981

[ptr70051-bib-0010] Al‐Rawashde, F. A. , A. S. Al‐Wajeeh , M. N. Vishkaei , et al. 2022. “Thymoquinone Inhibits JAK/STAT and PI3K/AKT/ MTOR Signaling Pathways in MV4‐11 and K562 Myeloid Leukemia Cells.” Pharmaceuticals 15, no. 9: 1123. 10.3390/ph15091123. 36145344 PMC9504933

[ptr70051-bib-0012] Aswathy, M. , D. Parama , M. Hegde , et al. 2024. “Natural Prenylflavones From the Stem Bark of *Artocarpus altilis* : Promising Anticancer Agents for Oral Squamous Cell Carcinoma Targeting the Akt/mTOR/STAT‐3 Signaling Pathway.” ACS Omega 9, no. 23: 24252–24267. 10.1021/acsomega.3c08376. 38882137 PMC11170706

[ptr70051-bib-0013] Battaglioni, S. , D. Benjamin , M. Wälchli , T. Maier , and M. N. Hall . 2022. “mTOR Substrate Phosphorylation in Growth Control.” Cell 185, no. 11: 1814–1836. 10.1016/j.cell.2022.04.013. 35580586

[ptr70051-bib-0014] Birgisdottir, Å. B. , S. Mouilleron , Z. Bhujabal , et al. 2019. “Members of the Autophagy Class III Phosphatidylinositol 3‐Kinase Complex I Interact With GABARAP and GABARAPL1 via LIR Motifs.” Autophagy 15, no. 8: 1333–1355. 30767700 10.1080/15548627.2019.1581009 PMC6613885

[ptr70051-bib-0015] Bouquier, N. , E. Moutin , L. A. Tintignac , et al. 2020. “Aimtor, a Bret Biosensor for Live Imaging, Reveals Subcellular Mtor Signaling and Dysfunctions.” BMC Biology 18, no. 1: 81. 10.1186/s12915-020-00790-8. 32620110 PMC7334845

[ptr70051-bib-0016] Boutouja, F. , C. M. Stiehm , and H. W. Platta . 2019. “mTOR: A Cellular Regulator Interface in Health and Disease.” Cells 8, no. 1: 18. 30609721 10.3390/cells8010018 PMC6356367

[ptr70051-bib-0017] Boya, P. , F. Reggiori , and P. Codogno . 2013. “Emerging Regulation and Functions of Autophagy.” Nature Cell Biology 15, no. 7: 713–720. 23817233 10.1038/ncb2788 PMC7097732

[ptr70051-bib-0018] Brockmueller, A. , S. Sameri , A. Liskova , et al. 2021. “Resveratrol's Anti‐Cancer Effects Through the Modulation of Tumor Glucose Metabolism.” Cancers 13, no. 2: 188. 10.3390/cancers13020188. 33430318 PMC7825813

[ptr70051-bib-0020] Chang, L. , P. H. Graham , J. Hao , et al. 2013. “Acquisition of Epithelial–Mesenchymal Transition and Cancer Stem Cell Phenotypes Is Associated With Activation of the PI3K/Akt/mTOR Pathway in Prostate Cancer Radioresistance.” Cell Death & Disease 4, no. 10: e875. 24157869 10.1038/cddis.2013.407 PMC3920940

[ptr70051-bib-0021] Chen, T. , J. Hao , J. He , et al. 2013. “Cannabisin B Induces Autophagic Cell Death by Inhibiting the AKT/mTOR Pathway and S Phase Cell Cycle Arrest in HepG2 Cells.” Food Chemistry 138, no. 2: 1034–1041. 23411211 10.1016/j.foodchem.2012.11.102

[ptr70051-bib-0023] Corti, F. , F. Nichetti , A. Raimondi , et al. 2019. “Targeting the PI3K/AKT/mTOR Pathway in Biliary Tract Cancers: A Review of Current Evidences and Future Perspectives.” Cancer Treatment Reviews 72: 45–55. 30476750 10.1016/j.ctrv.2018.11.001

[ptr70051-bib-0024] De Angel, R. E. , S. M. Smith , R. D. Glickman , S. N. Perkins , and S. D. Hursting . 2010. “Antitumor Effects of Ursolic Acid in a Mouse Model of Postmenopausal Breast Cancer.” Nutrition and Cancer 62, no. 8: 1074–1086. 21058195 10.1080/01635581.2010.492092

[ptr70051-bib-0025] Dibble, C. C. , and B. D. Manning . 2013. “Signal Integration by mTORC1 Coordinates Nutrient Input With Biosynthetic Output.” Nature Cell Biology 15, no. 6: 555–564. 23728461 10.1038/ncb2763 PMC3743096

[ptr70051-bib-0026] Elmore, S. 2007. “Apoptosis: A Review of Programmed Cell Death.” Toxicologic Pathology 35, no. 4: 495–516. 17562483 10.1080/01926230701320337 PMC2117903

[ptr70051-bib-0027] El‐Wetidy, M. , H. Helal , and M. Rady . 2024. “Urolithin A Targets Both PI3K/P‐AKT/MTOR and P‐C‐RAF/MEK/P‐ERK Signaling Pathways in Colorectal Cancer.” Egyptian Academic Journal of Biological Sciences. C, Physiology and Molecular Biology 16, no. 1: 387–401. 10.21608/eajbsc.2024.355267.

[ptr70051-bib-0028] Esmeeta, A. , S. Adhikary , V. Dharshnaa , et al. 2022. “Plant‐Derived Bioactive Compounds in Colon Cancer Treatment: An Updated Review.” Biomedicine & Pharmacotherapy 153: 113384. 35820317 10.1016/j.biopha.2022.113384

[ptr70051-bib-0029] Esteve, M. 2020. “Mechanisms Underlying Biological Effects of Cruciferous Glucosinolate‐Derived Isothiocyanates/Indoles: A Focus on Metabolic Syndrome.” Frontiers in Nutrition 7: 111. 10.3389/fnut.2020.00111. 32984393 PMC7492599

[ptr70051-bib-0033] Feitelson, M. A. , A. Arzumanyan , R. J. Kulathinal , et al. 2015. “Sustained Proliferation in Cancer: Mechanisms and Novel Therapeutic Targets.” Seminars in Cancer Biology 35: S25–S54. 10.1016/j.semcancer.2015.02.006. 25892662 PMC4898971

[ptr70051-bib-0034] Fernández‐Lázaro, D. , B. Sanz , and J. Seco‐Calvo . 2024. “Mechanisms of Programmed Cell Death: Structural and Functional Pathways. A Narrative Review.” Investigación Clínica 65, no. 2: 230–252. 10.54817/ic.v65n2a09. PMC10801515 38250814

[ptr70051-bib-0036] Ganesan, K. , C. Xu , J. Wu , et al. 2024. “Ononin Inhibits Triple‐Negative Breast Cancer Lung Metastasis by Targeting the EGFR‐Mediated PI3K/Akt/mTOR Pathway.” Science China Life Sciences 67, no. 9: 1849–1866. 10.1007/s11427-023-2499-2. 38900236

[ptr70051-bib-0037] Gao, J. H. , C. H. Wang , H. Tong , S. L. Wen , Z. Y. Huang , and C. W. Tang . 2015. “Targeting Inhibition of Extracellular Signal‐Regulated Kinase Kinase Pathway With AZD6244 (ARRY‐142886) Suppresses Growth and Angiogenesis of Gastric Cancer.” Scientific Reports 5, no. 1: 1–13. 10.1038/srep16382 PMC4644956 26567773

[ptr70051-bib-0038] Gao, Z. , B. Pang , J. Li , N. Gao , T. Fan , and Y. Li . 2021. “Emerging Role of Exosomes in Liquid Biopsy for Monitoring Prostate Cancer Invasion and Metastasis.” Frontiers in Cell and Developmental Biology 9: 679527. 10.3389/fcell.2021.679527. 34017837 PMC8129505

[ptr70051-bib-0039] Gill, B. S. , S. Kumar , and Navgeet 2016. “Triterpenes in Cancer: Significance and Their Influence.” Molecular Biology Reports 43, no. 9: 881–896. 10.1007/s11033-016-4032-9. 27344437

[ptr70051-bib-0040] Girodengo, M. , S. K. Ultanir , and J. M. Bateman . 2022. “Mechanistic Target of Rapamycin Signaling in Human Nervous System Development and Disease.” Frontiers in Molecular Neuroscience 15: 1005631. 10.3389/fnmol.2022.1005631. 36226315 PMC9549271

[ptr70051-bib-0041] Glaviano, A. , A. S. Foo , H. Y. Lam , et al. 2023. “PI3K/AKT/Mtor Signaling Transduction Pathway and Targeted Therapies in Cancer.” Molecular Cancer 22, no. 1: 138. 10.1186/s12943-023-01827-6. 37596643 PMC10436543

[ptr70051-bib-0042] Grabiner, B. C. , V. Nardi , K. Birsoy , et al. 2014. “A Diverse Array of Cancer‐Associated MTOR Mutations Are Hyperactivating and Can Predict Rapamycin Sensitivity.” Cancer Discovery 4, no. 5: 554–563. 24631838 10.1158/2159-8290.CD-13-0929 PMC4012430

[ptr70051-bib-0043] Gu, R. , M. Zhang , H. Meng , D. Xu , and Y. Xie . 2018. “Gallic Acid Targets Acute Myeloid Leukemia via Akt/mTOR‐Dependent Mitochondrial Respiration Inhibition.” Biomedicine & Pharmacotherapy 105: 491–497. 29883944 10.1016/j.biopha.2018.05.158

[ptr70051-bib-0044] Guo, R. , Q. Meng , H. Guo , et al. 2016. “TGF‐β2 Induces Epithelial‐Mesenchymal Transition in Cultured Human Lens Epithelial Cells Through Activation of the PI3K/Akt/mTOR Signaling Pathway.” Molecular Medicine Reports 13, no. 2: 1105–1110. 26647778 10.3892/mmr.2015.4645 PMC4732853

[ptr70051-bib-0045] Gupta, J. , A. Ahuja , and R. Gupta . 2022. “Green Approaches for Cancers Management: An Effective Tool for Health Care.” Anti‐Cancer Agents in Medicinal Chemistry 22, no. 1: 101–114. 33463475 10.2174/1871520621666210119091826

[ptr70051-bib-0046] Guru, S. K. , A. S. Pathania , S. Kumar , et al. 2015. “Secalonic Acid‐D Represses HIF1α/VEGF‐Mediated Angiogenesis by Regulating the Akt/mTOR/p70S6K Signaling CascadeSAD, a Mycotoxin Represses VEGF‐Arbitrated Angiogenesis.” Cancer Research 75, no. 14: 2886–2896. 10.1371/journal.pone.0088891. 25977334

[ptr70051-bib-0047] Hamzehzadeh, L. , S. L. Atkin , M. Majeed , A. E. Butler , and A. Sahebkar . 2018. “The Versatile Role of Curcumin in Cancer Prevention and Treatment: A Focus on PI3K/Akt Pathway.” Journal of Cellular Physiology 233, no. 10: 6530–6537. 10.1002/jcp.26620. 29693253

[ptr70051-bib-0048] Haroon, M. , and S. C. Kang . 2020. “Celastrol‐Mediated Autophagy Regulation in Cancer.” Applied Biological Chemistry 63, no. 1: 81. 10.1186/s13765-020-00565-3.

[ptr70051-bib-0049] Imran, M. , B. Salehi , J. Sharifi‐Rad , et al. 2019. “Kaempferol: A Key Emphasis to Its Anticancer Potential.” Molecules 24, no. 12: 2277. 10.3390/molecules24122277. 31248102 PMC6631472

[ptr70051-bib-0050] Into, T. , M. Inomata , E. Takayama , and T. Takigawa . 2012. “Autophagy in Regulation of Toll‐Like Receptor Signaling.” Cellular Signalling 24, no. 6: 1150–1162. 22333395 10.1016/j.cellsig.2012.01.020

[ptr70051-bib-0053] Jaglanian, A. , and E. Tsiani . 2020. “Rosemary Extract Inhibits Proliferation, Survival, Akt, and mTOR Signaling in Triple‐Negative Breast Cancer Cells.” International Journal of Molecular Sciences 21, no. 3: 810. 32012648 10.3390/ijms21030810 PMC7037743

[ptr70051-bib-0055] Jhanwar‐Uniyal, M. , S. L. Zeller , E. Spirollari , M. Das , S. J. Hanft , and C. D. Gandhi . 2024. “Discrete Mechanistic Target of Rapamycin Signaling Pathways, Stem Cells, and Therapeutic Targets.” Cells 13, no. 5: 409. 10.3390/cells13050409. 38474373 PMC10930964

[ptr70051-bib-0056] Jiang, B. H. , and L. Z. Liu . 2008. “AKT signaling in regulating angiogenesis.” Current Cancer Drug Targets 8, no. 1: 19–26. 18288940 10.2174/156800908783497122

[ptr70051-bib-0057] Jung, G. R. , K. J. Kim , C. H. Choi , et al. 2007. “Effect of Betulinic Acid on Anticancer Drug‐Resistant Colon Cancer Cells.” Basic & Clinical Pharmacology & Toxicology 101, no. 4: 277–285. 17845510 10.1111/j.1742-7843.2007.00115.x

[ptr70051-bib-0058] Khan, K. , C. Quispe , Z. Javed , et al. 2020. “Resveratrol, Curcumin, Paclitaxel and Mirnas Mediated Regulation of PI3K/AKT/mTOR Pathway: GO Four Better to Treat Bladder Cancer.” Cancer Cell International 20, no. 1: 560. 10.1186/s12935-020-01660-7. 33292283 PMC7685642

[ptr70051-bib-0059] Khan, M. A. , S. Siddiqui , I. Ahmad , et al. 2021. “Phytochemicals From Ajwa Dates Pulp Extract Induce Apoptosis in Human Triple‐Negative Breast Cancer by Inhibiting AKT/mTOR Pathway and Modulating Bcl‐2 Family Proteins.” Scientific Reports 11, no. 1: 1–14. 33990623 10.1038/s41598-021-89420-z PMC8121835

[ptr70051-bib-0061] Kim, J. , M. Kundu , B. Viollet , and K. L. Guan . 2011. “AMPK and mTOR Regulate Autophagy Through Direct Phosphorylation of Ulk1.” Nature Cell Biology 13, no. 2: 132–141. 21258367 10.1038/ncb2152 PMC3987946

[ptr70051-bib-0062] Kim, S. M. , P. Vetrivel , S. E. Ha , H. H. Kim , J. A. Kim , and G. S. Kim . 2020. “Apigetrin Induces Extrinsic Apoptosis, Autophagy and G2/M Phase Cell Cycle Arrest Through PI3K/AKT/mTOR Pathway in AGS Human Gastric Cancer Cell.” Journal of Nutritional Biochemistry 83: 108427. 32559585 10.1016/j.jnutbio.2020.108427

[ptr70051-bib-0063] Kimmelman, A. C. , and E. White . 2017. “Autophagy and Tumor Metabolism.” Cell Metabolism 25, no. 5: 1037–1043. 28467923 10.1016/j.cmet.2017.04.004 PMC5604466

[ptr70051-bib-0064] Kuballa, P. , W. M. Nolte , A. B. Castoreno , and R. J. Xavier . 2012. “Autophagy and the Immune System.” Annual Review of Immunology 30: 611–646. 10.1146/annurev-immunol-020711-074948 22449030

[ptr70051-bib-0065] Kumar, S. , and N. Agnihotri . 2019. “Piperlongumine, a Piper Alkaloid Targets Ras/PI3K/Akt/mTOR Signaling Axis to Inhibit Tumor Cell Growth and Proliferation in DMH/DSS Induced Experimental Colon Cancer.” Biomedicine & Pharmacotherapy 109: 1462–1477. 30551398 10.1016/j.biopha.2018.10.182

[ptr70051-bib-0066] Lamb, C. A. , T. Yoshimori , and S. A. Tooze . 2013. “The Autophagosome: Origins Unknown, Biogenesis Complex.” Nature Reviews Molecular Cell Biology 14, no. 12: 759–774. 24201109 10.1038/nrm3696

[ptr70051-bib-0067] Laplante, M. , and D. M. Sabatini . 2012. “mTOR Signaling in Growth Control and Disease.” Cell 149, no. 2: 274–293. 22500797 10.1016/j.cell.2012.03.017 PMC3331679

[ptr70051-bib-0068] Laplante, M. , and D. M. Sabatini . 2013. “Regulation of mTORC1 and Its Impact on Gene Expression at a Glance.” Journal of Cell Science 126, no. 8: 1713–1719. 23641065 10.1242/jcs.125773 PMC3678406

[ptr70051-bib-0069] Levenson, A. S. , and A. Kumar . 2020. “Pterostilbene as a Potent Chemopreventive Agent in Cancer.” In Natural Products for Cancer Chemoprevention, 49–108. Springer. 10.1007/978-3-030-39855-2_3.

[ptr70051-bib-0070] Li, X. , H. Liu , C. Lv , et al. 2022. “Gypenoside‐Induced Apoptosis via the PI3K/AKT/MTOR Signaling Pathway in Bladder Cancer.” BioMed Research International 2022: 1–15. 10.1155/2022/9304552. PMC8984741 35402614

[ptr70051-bib-0074] Lin, W. , S. Mehta , and J. Zhang . 2019. “Genetically Encoded Fluorescent Biosensors Illuminate Kinase Signaling in Cancer.” Journal of Biological Chemistry 294, no. 40: 14814–14822. 10.1074/jbc.rev119.006177. 31434714 PMC6779441

[ptr70051-bib-0075] Liu, F. , S. Gao , Y. Yang , et al. 2018. “Antitumor Activity of Curcumin by Modulation of Apoptosis and Autophagy in Human Lung Cancer A549 Cells Through Inhibiting PI3K/Akt/mTOR Pathway.” Oncology Reports 39, no. 3: 1523–1531. 29328421 10.3892/or.2018.6188

[ptr70051-bib-0076] Liu, M. , G. Zhao , D. Zhang , et al. 2018. “Active Fraction of Clove Induces Apoptosis via PI3K/Akt/mTOR‐Mediated Autophagy in Human Colorectal Cancer HCT‐116 Cells.” International Journal of Oncology 53, no. 3: 1363–1373. 30015913 10.3892/ijo.2018.4465

[ptr70051-bib-0077] Liu, Z. , M. Antalek , L. Nguyen , et al. 2013. “The Effect of Gartanin, a Naturally Occurring Xanthone in Mangosteen Juice, on the mTOR Pathway, Autophagy, Apoptosis, and the Growth of Human Urinary Bladder Cancer Cell Lines.” Nutrition and Cancer 65, no. sup1: 68–77. 23682785 10.1080/01635581.2013.785011 PMC3671488

[ptr70051-bib-0078] Liu, Z. , U. S. Ha , K. Yu , C. Wu , N. Yokoyama , and X. Zi . 2017. “Kavalactone Yangonin Induces Autophagy and Sensitizes Bladder Cancer Cells to Flavokawain A and Docetaxel via Inhibition of the mTOR Pathway.” Journal of Biomedical Research 31, no. 5: 408–418. 28959001 10.7555/JBR.31.20160160 PMC5706433

[ptr70051-bib-0080] Lunova, M. , B. Smolková , A. Lynnyk , et al. 2019. “Targeting the mTOR Signaling Pathway Utilizing Nanoparticles: A Critical Overview.” Cancers 11, no. 1: 82. 30642006 10.3390/cancers11010082 PMC6356373

[ptr70051-bib-0081] Mafi, S. , E. Ahmadi , E. Meehan , et al. 2023. “The mTOR Signaling Pathway Interacts With the ER Stress Response and the Unfolded Protein Response in Cancer.” Cancer Research 83, no. 15: 2450–2460. 10.1158/0008-5472.can-22-3032. 37195095

[ptr70051-bib-0082] Mafi, S. , B. Mansoori , S. Taeb , et al. 2022. “mTOR‐Mediated Regulation of Immune Responses in Cancer and Tumor Microenvironment.” Frontiers in Immunology 12: 5724. 10.3389/fimmu.2021.774103 PMC8894239 35250965

[ptr70051-bib-0083] Marafie, S. K. , F. Al‐Mulla , and J. Abubaker . 2024. “MTOR: Its Critical Role in Metabolic Diseases, Cancer, and the Aging Process.” International Journal of Molecular Sciences 25, no. 11: 6141. 10.3390/ijms25116141. 38892329 PMC11173325

[ptr70051-bib-0084] Mayer, I. A. , and C. L. Arteaga . 2016. “The PI3K/AKT Pathway as a Target for Cancer.” Annual Review of Medicine 67: 11–28. 10.1146/annurev-med-062913-051343 26473415

[ptr70051-bib-0086] Mehta, S. , and J. Zhang . 2021. “Biochemical Activity Architectures Visualized–Using Genetically Encoded Fluorescent Biosensors to Map the Spatial Boundaries of Signaling Compartments.” Accounts of Chemical Research 54, no. 10: 2409–2420. 10.1021/acs.accounts.1c00056. 33949851 PMC8580748

[ptr70051-bib-0087] Mihaylova, M. M. , and R. J. Shaw . 2011. “The AMPK Signaling Pathway Coordinates Cell Growth, Autophagy and Metabolism.” Nature Cell Biology 13, no. 9: 1016–1023. 21892142 10.1038/ncb2329 PMC3249400

[ptr70051-bib-0088] Mirza‐Aghazadeh‐Attari, M. , E. M. Ekrami , S. A. Aghdas , et al. 2020. “Targeting PI3K/AKT/Mtor Signaling Pathway by Polyphenols: Implication for Cancer Therapy.” Life Sciences 255: 117481. 10.1016/j.lfs.2020.117481. 32135183

[ptr70051-bib-0090] Moldogazieva, N. T. , S. P. Zavadskiy , and A. A. Terentiev . 2021. “Genomic Landscape of Liquid Biopsy for Hepatocellular Carcinoma Personalized Medicine.” Cancer Genomics Proteomics 18, no. 3: 369–383. 10.21873/cgp.20266. 33994362 PMC8240040

[ptr70051-bib-0092] Mori, S. , S. Nada , H. Kimura , et al. 2014. “The mTOR Pathway Controls Cell Proliferation by Regulating the FoxO_3_a mTOR Pathway and DNA Damage Response: A Therapeutic Strategy in Cancer.”

[ptr70051-bib-0094] Muhammad, N. , R. Steele , T. S. Isbell , N. Philips , and R. B. Ray . 2017. “Bitter Melon Extract Inhibits Breast Cancer Growth in Preclinical Model by Inducing Autophagic Cell Death.” Oncotarget 8, no. 39: 66226–66236. 29029506 10.18632/oncotarget.19887 PMC5630406

[ptr70051-bib-0095] Murugan, A. K. 2019. “mTOR: Role in Cancer, Metastasis and Drug Resistance.” In Seminars in Cancer Biology, vol. 59, 92–111. Academic Press. 31408724 10.1016/j.semcancer.2019.07.003

[ptr70051-bib-0096] Narayanankutty, A. 2021. “Inhibitory Potential of Dietary Nutraceuticals on Cellular PI3K/Akt Signaling: Implications in Cancer Prevention and Therapy.” Current Topics in Medicinal Chemistry 21, no. 20: 1816–1831. 10.2174/1568026621666210716152224. 34279200

[ptr70051-bib-0097] Nel, A. E. , L. Mädler , D. Velegol , et al. 2009. “Understanding Biophysicochemical Interactions at the Nano–Bio Interface.” Nature Materials 8, no. 7: 543–557. 19525947 10.1038/nmat2442

[ptr70051-bib-0099] Parveen, S. , R. Misra , and S. K. Sahoo . 2012. “Nanoparticles: a Boon to Drug Delivery, Therapeutics, Diagnostics and Imaging.” Nanomedicine: Nanotechnology, Biology and Medicine 8, no. 2: 147–166. 21703993 10.1016/j.nano.2011.05.016

[ptr70051-bib-0100] Pazhooh, R. D. , P. R. Farnood , Z. Asemi , L. Mirsafaei , B. Yousefi , and H. Mirzaei . 2021. “mTOR Pathway and DNA Damage Response: A Therapeutic Strategy in Cancer Therapy.” DNA Repair 104: 103142. 34102579 10.1016/j.dnarep.2021.103142

[ptr70051-bib-0101] Pistritto, G. , D. Trisciuoglio , C. Ceci , A. Garufi , and G. D'Orazi . 2016. “Apoptosis as Anticancer Mechanism: Function and Dysfunction of Its Modulators and Targeted Therapeutic Strategies.” Aging 8, no. 4: 603–619. 27019364 10.18632/aging.100934 PMC4925817

[ptr70051-bib-0102] Putcha, G. V. , C. A. Harris , K. L. Moulder , R. M. Easton , C. B. Thompson , and E. M. Johnson Jr. 2002. “Intrinsic and Extrinsic Pathway Signaling During Neuronal Apoptosis: Lessons From the Analysis of Mutant Mice.” Journal of Cell Biology 157, no. 3: 441–453. 11980919 10.1083/jcb.200110108 PMC2173286

[ptr70051-bib-0103] Rauf, A. , T. Abu‐Izneid , A. A. Khalil , et al. 2021. “Berberine as a Potential Anticancer Agent: A Comprehensive Review.” Molecules 26, no. 23: 7368. 10.3390/molecules26237368. 34885950 PMC8658774

[ptr70051-bib-0104] Rengarajan, T. , and N. S. Yaacob . 2016. “The Flavonoid Fisetin as an Anticancer Agent Targeting the Growth Signaling Pathways.” European Journal of Pharmacology 789: 8–16. 10.1016/j.ejphar.2016.07.001. 27377217

[ptr70051-bib-0105] Russell, R. C. , H. X. Yuan , and K. L. Guan . 2014. “Autophagy Regulation by Nutrient Signaling.” Cell Research 24, no. 1: 42–57. 24343578 10.1038/cr.2013.166 PMC3879708

[ptr70051-bib-0107] Saxton, R. A. , and D. M. Sabatini . 2017. “mTOR Signaling in Growth, Metabolism, and Disease.” Cell 168, no. 6: 960–976. 28283069 10.1016/j.cell.2017.02.004 PMC5394987

[ptr70051-bib-0108] Seok, J.‐W. , H.‐H. Lee , and K.‐H. Oh . 2019. “Antibacterial and Proteomic Effects of *Legionella pneumophila* JK‐3 Exposed to Green Tea Catechin, Epigallocatechin Gallate (EGCG).” KSBB Journal 34, no. 1: 15–24. 10.7841/ksbbj.2019.34.1.15.

[ptr70051-bib-0109] Sharifi‐Rad, J. , C. Quispe , M. Imran , et al. 2021. “Genistein: An Integrative Overview of Its Mode of Action, Pharmacological Properties, and Health Benefits.” Oxidative Medicine and Cellular Longevity 202: 1–36. 10.1155/2021/3268136. PMC8315847 34336089

[ptr70051-bib-0111] Singh, B. N. , S. Shankar , and R. K. Srivastava . 2011. “Green Tea Catechin, Epigallocatechin‐3‐Gallate (EGCG): Mechanisms, Perspectives and Clinical Applications.” Biochemical Pharmacology 82, no. 12: 1807–1821. 10.1016/j.bcp.2011.07.093. 21827739 PMC4082721

[ptr70051-bib-0112] Swiech, L. , M. Perycz , A. Malik , and J. Jaworski . 2008. “Role of mTOR in Physiology and Pathology of the Nervous System.” Biochimica et Biophysica Acta (BBA)‐Proteins and Proteomics 1784, no. 1: 116–132. 17913600 10.1016/j.bbapap.2007.08.015

[ptr70051-bib-0113] Tao, J. , Y. Li , S. Li , and H. B. Li . 2018. “Plant Foods for the Prevention and Management of Colon Cancer.” Journal of Functional Foods 42: 95–110.

[ptr70051-bib-0114] Tey, S. K. , and R. Khanna . 2012. “Host Immune System Strikes Back: Autophagy‐Mediated Antigen Presentation Bypasses Viral Blockade of the Classic MHC Class I Processing Pathway.” Autophagy 8, no. 12: 1839–1841. 22932396 10.4161/auto.21860 PMC3541298

[ptr70051-bib-0115] Thomson, A. W. , H. R. Turnquist , and G. Raimondi . 2009. “Immunoregulatory Functions of mTOR Inhibition.” Nature Reviews Immunology 9, no. 5: 324–337. 10.1038/nri2546 PMC2847476 19390566

[ptr70051-bib-0117] Tlili, H. , A. Macovei , D. Buonocore , et al. 2021. “The Polyphenol/Saponin‐Rich *Rhus tripartita* Extract Has an Apoptotic Effect on THP‐1 Cells Through the PI3K/AKT/mTOR Signaling Pathway.” BMC Complementary Medicine and Therapies 21, no. 1: 1–13. 34044827 10.1186/s12906-021-03328-9 PMC8161611

[ptr70051-bib-0118] Tsai, J. P. , C. H. Lee , T. H. Ying , et al. 2015. “Licochalcone A Induces Autophagy Through PI3K/Akt/mTOR Inactivation and Autophagy Suppression Enhances Licochalcone A‐Induced Apoptosis of Human Cervical Cancer Cells.” Oncotarget 6, no. 30: 28851–28866. 26311737 10.18632/oncotarget.4767 PMC4745696

[ptr70051-bib-0119] Tsai, Y. P. , and K. J. Wu . 2012. “Hypoxia‐Regulated Target Genes Implicated in Tumor Metastasis.” Journal of Biomedical Science 19, no. 1: 1–7. 23241400 10.1186/1423-0127-19-102 PMC3541338

[ptr70051-bib-0120] Wang, C. Z. , Z. Zhang , J. Y. Wan , et al. 2015. “Protopanaxadiol, an Active Ginseng Metabolite, Significantly Enhances the Effects of Fluorouracil on Colon Cancer.” Nutrients 7, no. 2: 799–814. 25625815 10.3390/nu7020799 PMC4344561

[ptr70051-bib-0122] Wang, R. , X. Lu , and R. Yu . 2020. “Lycopene Inhibits Epithelial–Mesenchymal Transition and Promotes Apoptosis in Oral Cancer via PI3K/AKT/m‐TOR Signal Pathway.” Drug Design, Development and Therapy 14: 2461–2471. 32606612 10.2147/DDDT.S251614 PMC7321693

[ptr70051-bib-0124] Watanabe, R. , L. Wei , and J. Huang . 2011. “mTOR Signaling, Function, Novel Inhibitors, and Therapeutic Targets.” Journal of Nuclear Medicine 52, no. 4: 497–500. 21421716 10.2967/jnumed.111.089623

[ptr70051-bib-0125] Weichhart, T. , M. Hengstschläger , and M. Linke . 2015. “Regulation of Innate Immune Cell Function by mTOR.” Nature Reviews Immunology 15, no. 10: 599–614. 10.1038/nri3901 PMC6095456 26403194

[ptr70051-bib-0126] White, E. 2015. “The Role for Autophagy in Cancer.” Journal of Clinical Investigation 125, no. 1: 42–46. 25654549 10.1172/JCI73941 PMC4382247

[ptr70051-bib-0127] Wu, H. , W. Lai , Q. Wang , Q. Zhou , R. Zhang , and Y. Zhao . 2024. “Gypenoside Induces Apoptosis by Inhibiting the PI3K/AKT/mTOR Pathway and Enhances T‐Cell Antitumor Immunity by Inhibiting PD‐L1 in Gastric Cancer.” Frontiers in Pharmacology 15: 1243353. 10.3389/fphar.2024.1243353. 38482051 PMC10933075

[ptr70051-bib-0129] Xia, J. , S. Guo , T. Fang , et al. 2014. “Dihydromyricetin Induces Autophagy in HepG2 Cells Involved in Inhibition of mTOR and Regulating Its Upstream Pathways.” Food and Chemical Toxicology 66: 7–13. 24444546 10.1016/j.fct.2014.01.014

[ptr70051-bib-0130] Xie, H. , J. Rutz , S. Maxeiner , et al. 2022. “Plant‐Derived Sulforaphane Suppresses Growth and Proliferation of Drug‐Sensitive and Drug‐Resistant Bladder Cancer Cell Lines In Vitro.” Cancers 14, no. 19: 4682. 10.3390/cancers14194682. 36230603 PMC9564120

[ptr70051-bib-0131] Yu, C. H. , C. Z. Wang , and C. S. Yuan . 2013. “Progress in Anti‐Cancer Research of American Ginseng: With an Example of Colorectal Cancer.” Acta Pharmaceutica Sinica 48, no. 7: 986–992. 24133965

[ptr70051-bib-0132] Yu, Y. , S.‐O. Yoon , G. Poulogiannis , et al. 2011. “Phosphoproteomic Analysis Identifies grb10 as an mtorc1 Substrate That Negatively Regulates Insulin Signaling.” Science 332, no. 6035: 1322–1326. 10.1126/science.1199484. 21659605 PMC3195509

[ptr70051-bib-0133] Yun, H. R. , Y. H. Jo , J. Kim , Y. Shin , S. S. Kim , and T. G. Choi . 2020. “Roles of Autophagy in Oxidative Stress.” International Journal of Molecular Sciences 21, no. 9: 3289. 32384691 10.3390/ijms21093289 PMC7246723

[ptr70051-bib-0134] Zhang, F. , T. Cheng , and S.‐X. Zhang . 2023. “Mechanistic Target of Rapamycin (mTOR): A Potential New Therapeutic Target for Rheumatoid Arthritis.” Arthritis Research & Therapy 25, no. 1: 187. 10.1186/s13075-023-03181-w. 37784141 PMC10544394

[ptr70051-bib-0135] Zhang, H. W. , J. J. Hu , R. Q. Fu , et al. 2018. “Flavonoids Inhibit Cell Proliferation and Induce Apoptosis and Autophagy Through Downregulation of PI3Kγ Mediated PI3K/AKT/mTOR/p70S6K/ULK Signaling Pathway in Human Breast Cancer Cells.” Scientific Reports 8, no. 1: 1–13. 30050147 10.1038/s41598-018-29308-7 PMC6062549

[ptr70051-bib-0136] Zhang, P. , Z. L. Lai , H. F. Chen , et al. 2017. “Curcumin Synergizes With 5‐Fluorouracil by Impairing AMPK/ULK1‐Dependent Autophagy, AKT Activity and Enhancing Apoptosis in Colon Cancer Cells With Tumor Growth Inhibition in Xenograft Mice.” Journal of Experimental & Clinical Cancer Research 36: 1–12. 29273065 10.1186/s13046-017-0661-7 PMC5741949

[ptr70051-bib-0137] Zhao, Q. , C. Peng , C. Zheng , X.‐H. He , W. Huang , and B. Han . 2020. “Recent Advances in Characterizing Natural Products That Regulate Autophagy.” Anti‐Cancer Agents in Medicinal Chemistry 19, no. 18: 2177–2196. 10.2174/1871520619666191015104458. 31749434

[ptr70051-bib-0138] Zhou, C. , J. U. N. Ding , and Y. Wu . 2014. “Resveratrol Induces Apoptosis of Bladder Cancer Cells via miR‐21 Regulation of the Akt/Bcl‐2 Signaling Pathway.” Molecular Medicine Reports 9, no. 4: 1467–1473. 24535223 10.3892/mmr.2014.1950

[ptr70051-bib-0139] Zhou, J. , Z. Guo , X. Peng , et al. 2024. “Chrysotoxine Regulates Ferroptosis and the PI3K/AKT/mTOR Pathway to Prevent Cervical Cancer.” Journal of Ethnopharmacology 338: 119126. 10.1016/j.jep.2024.119126. 39557107

[ptr70051-bib-0140] Zhou, R. , H. Chen , J. Chen , X. Chen , Y. Wen , and L. Xu . 2018. “Extract From Astragalus Membranaceus Inhibit Breast Cancer Cells Proliferation via PI3K/AKT/mTOR Signaling Pathway.” BMC Complementary and Alternative Medicine 18: 1–8. 29523109 10.1186/s12906-018-2148-2 PMC5845298

[ptr70051-bib-0141] Zhou, X. , T. L. Clister , P. R. Lowry , M. M. Seldin , G. W. Wong , and J. Zhang . 2015. “Dynamic Visualization of mtorc1 Activity in Living Cells.” Cell Reports 10, no. 10: 1767–1777. 10.1016/j.celrep.2015.02.031. 25772363 PMC4567530

[ptr70051-bib-0142] Zhu, J. , and C. B. Thompson . 2019. “Metabolic Regulation of Cell Growth and Proliferation.” Nature Reviews Molecular Cell Biology 20, no. 7: 436–450. 30976106 10.1038/s41580-019-0123-5 PMC6592760

[ptr70051-bib-0143] Zhu, Y. , N. Xie , Y. Chai , et al. 2022. “Apoptosis Induction, a Sharp Edge of Berberine to Exert Anti‐Cancer Effects, Focus on Breast, Lung, and Liver Cancer.” Frontiers in Pharmacology 13: 803717. 10.3389/fphar.2022.803717. 35153781 PMC8830521

[ptr70051-bib-0144] Zuo, M. , H. Chen , Y. Liao , et al. 2023. “Sulforaphane and Bladder Cancer: a Potential Novel Antitumor Compound.” Frontiers in Pharmacology 14: 1254236. 10.3389/fphar.2023.1254236. 37781700 PMC10540234

