# Supplementary material for: Current Insights of the Potential Plant Bioactive Compounds on Modulating the mTOR Signaling Pathway in Regulating Oncological Disorders
Source: Phytother Res. 2025 Jul 27;39(9):4046–59. doi: 10.1002/ptr.70051 (PMC12423487; doi:10.1002/ptr.70051)
Supplement: Supplementary file 1 — Data S1. Supporting Information. [file PTR-39-4046-s001.docx]

- **Abbreviation**
  - 4EBP1: Eukaryotic Translation Initiation Factor 4E-Binding Protein 1
  - AKT/PKB: Protein Kinase B
  - AMBRA1: Activating Molecule in Beclin1-Regulated Autophagy
  - AMPK: AMP-activated Protein Kinase
  - Apaf1: Apoptotic Protease Activating Factor 1
  - ATG: Autophagy-related proteins
  - ATG101: Autophagy-related protein 101
  - ATG13: Autophagy-related protein 13
  - ATG3/7: Autophagy-related protein 3/7
  - Bax: Bcl-2-associated X protein
  - BCL: B-cell lymphoma
  - Bcl-2: B-cell lymphoma 2
  - Bcl-xL: B-cell lymphoma-extra large
  - BH3: Bcl-2 Homology 3
  - BID: BH3 Interacting Domain Death Agonist
  - Beclin-1: Autophagy-related protein 6
  - Caspases: Cysteine-aspartic proteases
  - DEPTOR: DEP domain-containing mTOR-interacting protein
  - DRS: Death Receptor Signaling
  - EGFR/HER2: Epidermal Growth Factor Receptor/Human Epidermal Growth Factor Receptor 2
  - EBPI: Erythrocyte Band 4.1-like Protein 1
  - ERK: Extracellular Signal-Regulated Kinase
  - FADD: Fas-associated death domain
  - Fas: Fatty acid synthase
  - FIP200: FAK Family Kinase Interacting Protein of 200 kDa
  - FOXO: Forkhead Box O
  - GDP Rheb: Guanosine Diphosphate-bound Rheb
  - GLUT1: Glucose Transporter 1
  - Grb2: Growth Factor Receptor-bound protein 2
  - IEGSK3: Immediate Early Response Gene 2
  - IGFR: Insulin-like Growth Factor Receptor
  - IRS: Insulin Receptor Substrate
  - LKB1: Liver Kinase B1
  - LC3: Microtubule-associated protein 1A/1B-light chain 3
  - Lipin 1: Phosphatidate Phosphatase LPIN1
  - MCL: Myeloid cell leukemia
  - MEK: Mitogen-Activated Protein Kinase
  - MDM2: Mouse Double Minute 2 homolog
  - mLST8: Mammalian Lethal with SEC13 protein 8
  - mTOR: Mammalian Target of Rapamycin
  - MSI1: Musashi RNA-binding protein 1
  - PARP: Poly (ADP-ribose) polymerase
  - PDGFR: Platelet-Derived Growth Factor Receptor
  - PI3K: Phosphoinositide-3-kinase
  - P110, p85: Subunits of phosphatidylinositol 3-kinase-alpha
  - P27: Cyclin-dependent kinase inhibitor 1B
  - P53: Tumor Protein p53
  - PKC1: Protein Kinase C 1
  - PKCθ: Protein Kinase C theta
  - PRAS40: Proline-Rich AKT Substrate of 40 kDa
  - PTEN: Phosphatase and Tensin homolog
  - Rheb: Ras homolog enriched in brain
  - Raptor: Regulatory-Associated Protein of mTOR
  - RAS: Rat Sarcoma
  - RAF: Rapidly Accelerated Fibrosarcoma
  - Rictor: Rapamycin-Insensitive Companion of mTOR
  - ROS: Reactive Oxygen Species
  - RTK: Receptor Tyrosine Kinases
  - SGK1: Serum and Glucocorticoid-regulated Kinase 1
  - S6K: p70 Ribosomal S6 Kinase
  - SGK1: Serum and Glucocorticoid-regulated Kinase 1
  - SIRT1: Sirtuin 1
  - Smac: Second Mitochondria-derived Activator of Caspase
  - TLR4: Toll-like Receptor 4
  - TNFRSF1A: Tumor Necrosis Factor Receptor Superfamily Member 1A
  - TRADD: TNFRSF1A-associated via death domain
  - TRAILR: Tumor Necrosis Factor Receptor Superfamily Member 10A
  - TSC1/2: Tuberous Sclerosis Complex ½
  - TTF1: Thyroid Transcription Factor 1
  - ULK1/2: Unc-51 Like Autophagy Activating Kinase ½
  - VEGFR: Vascular Endothelial Growth Factor Receptor
  - VPS15: Vacuolar Protein Sorting 15
  - VPS34: Vacuolar Protein Sorting 34
  - XIAP: X-linked Inhibitor of Apoptosis Protein
